# African swine fever virus genes vectored by simian adenoviruses do not protect against virulent genotype II virus challenge

**DOI:** 10.1128/spectrum.02328-25

**Published:** 2026-02-27

**Authors:** Priscilla Y. L. Tng, Laila Al-Adwani, Lynnette C. Goatley, Eleni-Anna Loundras, Claire Powers, Christopher L. Netherton

**Affiliations:** 1 The Pirbright Institute, Woking, United Kingdom; 2 Pandemic Sciences Institute, Oxford, United Kingdom; National Microbiology Laboratory, Winnipeg, Manitoba, Canada

**Keywords:** pig, adenovirus, vaccine, African swine fever virus

## Abstract

African swine fever virus (ASFV) causes a lethal hemorrhagic disease in domestic pigs, wild boar, and other members of the genus *Sus*. Since the introduction of the virus into Georgia in 2007, the disease has spread across Eurasia causing the deaths of millions of animals and the production loss of more than 100 million with a significant socioeconomic impact. Modified live virus vaccines have been licensed and tested in several countries, but there are some residual safety concerns and the efficacy in the field is yet to be established. A subunit vaccine would provide a significant biosafety advantage and avoid the use of live African swine fever virus and would enable deployment of a simple distinguish infected from vaccinated animals (DIVA) strategy. However, the development of effective subunit vaccines has been slow due in part to the complexity of the virus and a poor understanding of the protective immune response. A pool of six viral genes vectored using replication-deficient adenoviruses could protect pigs against genotype I challenge; however, a combination of vectors partially tailored to genotype II ASFV was not successful after a genotype II challenge. Here, we provide immunogenicity and challenge data from a study using genotype II ASFV genes vectored using replication-deficient simian adenoviruses. The six antigens previously shown to protect against severe disease caused by genotype I challenge were supplemented with additional antigens, but these were not sufficient to prevent clinical disease after challenge with genotype II ASFV.

**IMPORTANCE:**

African swine fever is a viral hemorrhagic disease of domestic and wild pigs that poses a significant threat to farmers and global food security. Millions of animals have been lost due to the disease itself, control measures, and the knock-on effect on the pig production chain. African swine fever is also a conservation risk as it infects and kills endangered indigenous pigs in southern and eastern Asia and Oceania. Disease control efforts are in part frustrated by the lack of a safe and effective vaccine. Modified live virus vaccines are being deployed in select countries; however, a subunit vaccine like those developed for COVID would alleviate safety concerns with using live viruses in the field. We tested a combination of eight different virus genes delivered using replication-deficient adenoviruses and showed that they induced immune responses in pigs but did not lead to protection against disease.

## INTRODUCTION

African swine fever (ASF) is a virulent viral disease of domestic pigs with a high case fatality rate that was introduced into the country of Georgia in 2007. Since then, the disease has spread across Eurasia and has been reported in Haiti, Dominican Republic, and Papua New Guinea. ASF can be devastating to pig farmers, millions of pigs have been killed by the disease or in efforts to control it, and the Chinese herd was reduced by tens of millions of animals during the peak of disease in 2019. Although Belgium, Czechia, and Sweden have successfully contained and eradicated isolated outbreaks of the disease, most affected countries are suffering endemicity in domestic pigs and/or wild boar. ASF can be pernicious depending on the epidemiological context, for example, the disease was persistent on the island of Sardinia for over 45 years after its introduction during a preceding European epizootic between 1960 and 1999. The principal tool missing from disease control measures is a safe and effective vaccine. Modified live virus (MLV) vaccines based on the Georgia 2007 backbone have shown promise, but safety concerns remain principally around transmission in the field (1, 2), reversion to virulence (3, 4), and an inability to differentiate vaccinated animals from naturally infected ones. Unfortunately, these MLV vaccines do not protect against all isolates currently circulating in Asia, further limiting their utility (5). A second-generation, or subunit vaccine, would alleviate many of the concerns around MLV vaccines; however, the state of the art is currently significantly behind that of MLVs, with few research groups reporting successful results in challenge experiments (6–13).

A major hurdle to the development of an ASF subunit vaccine is the complexity of the virus, both in terms of structure and genomic content. African swine fever virus (ASFV) is a large double-stranded DNA virus with a genome length of between 170,000 and 194,000 base pairs. ASFV genotypes have been classified based on a 400 bp section of the 3′ end of the *B646L* gene that encodes for the major capsid protein p72 and using this approach a total of 23 ASFV genotypes (numbered I through XVII, and XIX through XXIV) have been identified to date (14–18), as well as, a hybrid recombinant derived from genotypes I and II (19, 20). The current panzootic is caused by a genotype II virus, and the previous panzootic that occurred between 1960 and 1999 was caused by a genotype I virus. More recent work has typed ASFV based on the complete sequence of the p72 protein (21) or used machine learning to assign “biotypes” based on the complete genome sequence (22). Georgia 2007/1 and related viruses are classified as 3′ *B646L* genotype II, p72 genotype 2 and biotype 2, while viruses related to the previous panzootic are 3′ *B646L* genotype 1, p72 genotype 1 and biotype 1.

Virions have a complex architecture comprising the nucleoid and core shell which is surrounded by an inner envelope upon which the capsid is formed (23–25). Virions obtain an additional external envelope after budding from cells, and both intracellular and extracellular virions are infectious (26). Virions contain more than 60 viral proteins (27), and the viral genome may encode for up to 250 different open reading frames that could be considered for incorporation into a vaccine (28). ASF convalescent pigs have virus-specific antibody and cellular immune responses, and experiments with genotype I ASFV have implicated both having a role in protection against disease (29–31).

Antibody-dependent cellular cytotoxicity (32), complement-dependent cytotoxicity (33), virus neutralizing (34–39), virus neutralizing inhibiting (36), hemadsorption inhibition (40, 41), and infection-enhancing activities (7, 42) have been described in serum from convalescent or vaccinated animals. Demonstrating neutralization of ASFV *in vitro* is technically challenging (39, 43), and a neutralizing antibody response *per se* does not prevent disease (44). Blocking virus entry might be feasible as antibodies against p54 (*E183L* gene), p30 (*CP204L* gene), and p72 (*B646L* gene) have all been implicated in neutralization (38, 45) and pO61R (p12) is involved in virus attachment (46, 47). All of these proteins are components or are predicted to be components of the intracellular particle. The external envelope is less well understood as the only confirmed viral component is pEP402R (CD2v), which is similar to the cellular CD2 protein and encoded by the *EP402R* gene (27). pEP402R is responsible for the hemadsorption of red blood cells to infected macrophages (48) and antibodies that block hemadsorption have been used to describe at least nine different immunotypes (49). ASFV immunotypes can be predicted using the sequence of pEP402R along with the c-type lectin encoded by the *EP153R* gene (41, 50); however, MLVs lacking these genes do offer protection against disease against virulent challenge (3, 51). pEP402R and pEP153R are also T-cell antigens (52), and depletion of CD8α^+^ cells abrogates protection against disease afforded by low virulent genotype I OUR/T1988/3 against homologous virulent ASFV, suggesting an important role for cellular immunity (31). Virus-specific interferon gamma (IFNγ) and tumor necrosis factor (TNF) alpha secreting cells (53–56), and ASFV-specific cytolytic activities have been identified (57, 58); however, the specific role of these activities is unknown. Therefore, it is likely that an effective ASFV subunit vaccine will need to induce virus-specific antibody and cellular immune responses, although DNA vaccination experiments have demonstrated partial protection against genotype I ASFV in the absence of detectable antibody responses (59).

Replication-deficient adenoviruses (rAds) induce robust humoral and cellular immune responses to transgenes and human adenovirus 5 (HuAd5), human adenovirus 2 (HuAd2), and a modified chimpanze adenovirus (ChAdOx1) have been successfully used in immunization experiments with ASFV genes (8, 10–13, 60–65). Immunization with HuAd5 followed by boost with modified vaccinia Ankara (MVA) vectors expressing *B602L*, *B646L*, *CP204L*, *E183L*, *E199L*, *EP153R*, *F317L*, and *MGF505-5R* prevented severe disease in domestic pigs after challenge with genotype I isolate OUR T1988/1 (10). Subsequent experiments demonstrated that heterologous MVA boost could be substituted with homologous rAd boost and that *B646L* and *CP204L* were not required for protection against genotype I ASFV (13). However, a combination of rAd vectored genotype I *E199L*, *F317L*, and *MGF505-5R* and genotype II *B602L*, *E183L*, and *EP153R* was not able to protect pigs against genotype II Georgia 2007/1 challenge. This combination of antigens induced humoral immune responses in domestic pigs, but cellular responses against genotype II virus were poor. Clinical signs and viremia were delayed after challenge with Georgia 2007/1, which suggests that the vaccine-induced antibody response was insufficient to completely control virus replication. These data suggested that additional antigens may be required to achieve protection against genotype II ASFV. We hypothesized that adding ASFV antigens pO61R and pEP402R that are involved in virus attachment may enhance the antibody responses against genotype II ASFV. In addition, broadening the T-cell response to include other T-cell antigens identified by screening cells from recovered animals using whole-proteome or targeted approaches (11, 62, 66, 67) may boost the poor genotype II T-cell response observed previously (13). DNA vaccination experiments have shown the utility of ubiquitinylated fusion proteins as an effective mechanism of delivering protective T-cell responses against ASFV (59, 68). Here, we present immunization and challenge data with a pool of eight ChAdOx1 vectored ASFV gene supplemented with ChAdOx1 vectored ubiquitinylated fusion protein containing a number of previously identified T-cell antigens.

## RESULTS

Individual ChAdOx1 vectors expressing genotype II versions of six ASFV genes that prevented severe disease against genotype I ASFV, *B602L*, *E183L*, *E199L*, *EP153R*, *F317L*, and *MGF505-5R* were generated. Two additional ChAdOx1 vectors expressing the cell surface and outer envelope pEP402R (CD2v) or the pO61R (p12) attachment protein were also generated. Finally, to try and increase the breadth of the virus-specific T-cell response, ubiquitin, *I73R*, *F334L*, *CP204L*, *M448R*, and *B646L* were fused together in a single open reading frame (ubiquitinylated polyprotein) and incorporated into a single ChAdOx1 vector. Cellular immune responses against these proteins had been identified in previous data (62, 66) and a group of pigs immunized with pools of adenoviruses that included those expressing *I73R*, *CP204L*, *M448R*, and *B646L* induced weak protective responses (62). Adenovirus transgene expression was confirmed by indirect immunofluorescence using anti-sera from recovered pigs (Fig. S1) and hemadsorption of red blood cells to cells infected with ChAdOx1-EP402R (Fig. S2).

Groups of six pigs were then immunized with a control ChAdOx1 expressing green fluorescent protein (GFP), a pool of the eight ChAdOx1 expressing *B602L*, *E183L*, *E199L*, *EP153R*, *EP402R*, *F317L*, *O61R*, and *MGF505-5R* or a pool comprised of the same eight ChAdOx1 vectors in combination with a vector expressing the ubiquitinylated polyprotein. Animals were inoculated via the intramuscular route with a dose of 5 × 10^9^ infectious units of each vector and then boosted 4 weeks later with the same combination and doses of ChAdOx1 at the same site (Fig. S3). No adverse effects were observed in any of the pigs, suggesting that the vectors were well tolerated.

**Fig 1:**
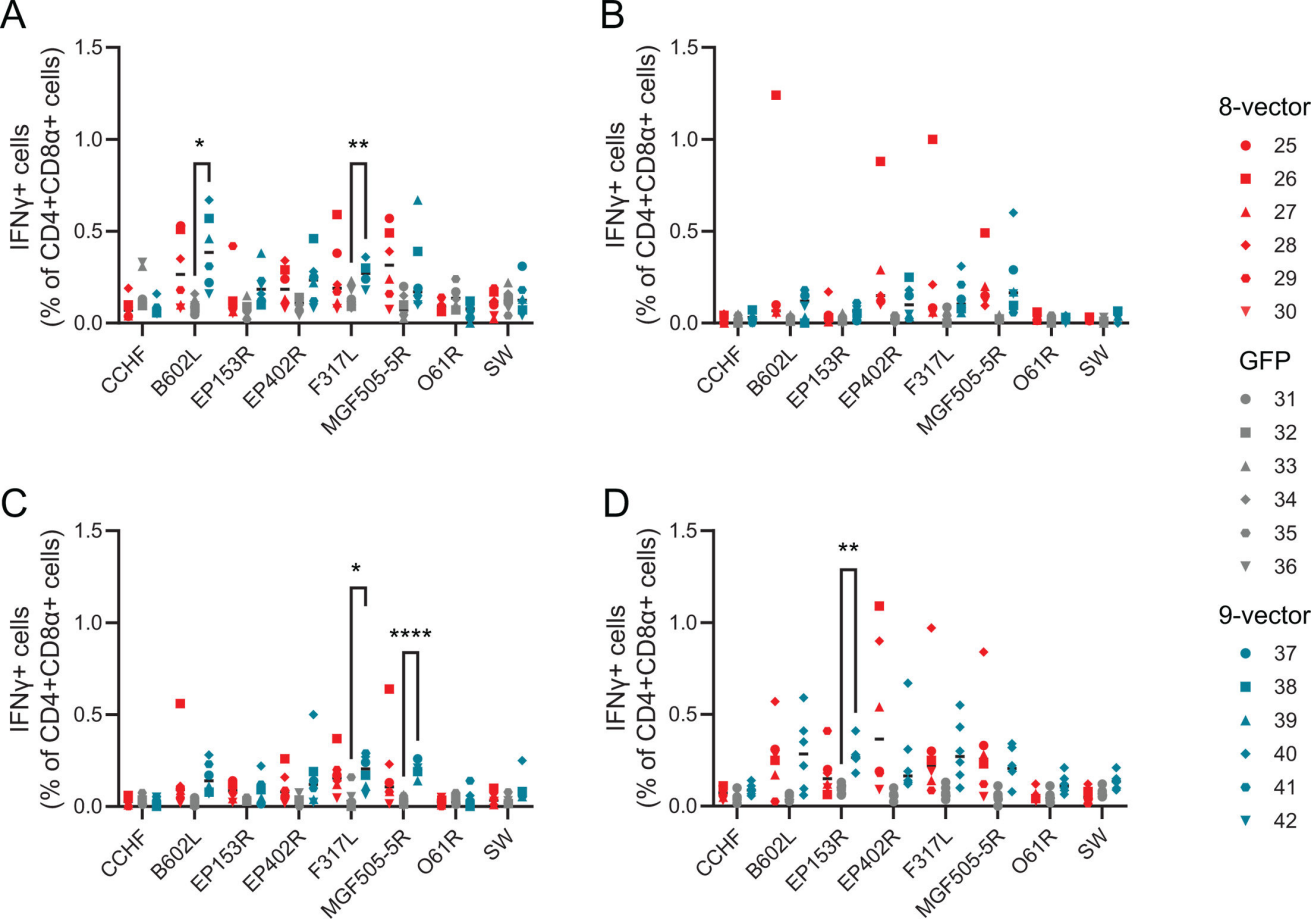
Antigen-specific CD4 T-cell responses. Animals were immunized with eight vectors (red), GFP (gray) or nine vectors (blue), boosted 28 days later and then challenged on Day 52. Cells were purified from blood collected on the indicated days and stimulated with peptide pools corresponding to the indicated gene products or the ubiquitinylated polyprotein (SW). Populations of CD3^+^CD4^+^CD8α^+^ cells were identified by flow cytometry and the proportion of them expressing IFNγ on 2 weeks post-prime (**A**), pre-boost (**B**), 7 days post-boost (**C**), and pre-challenge (**D**). Each data point indicates a single animal, and bars show the mean of each group. Green fluorescent protein (GFP); interferon gamma (IFNγ). *P ≤ 0.05, ** ≤ 0.01, and ****P ≤ 0.0001.

**Fig 2:**
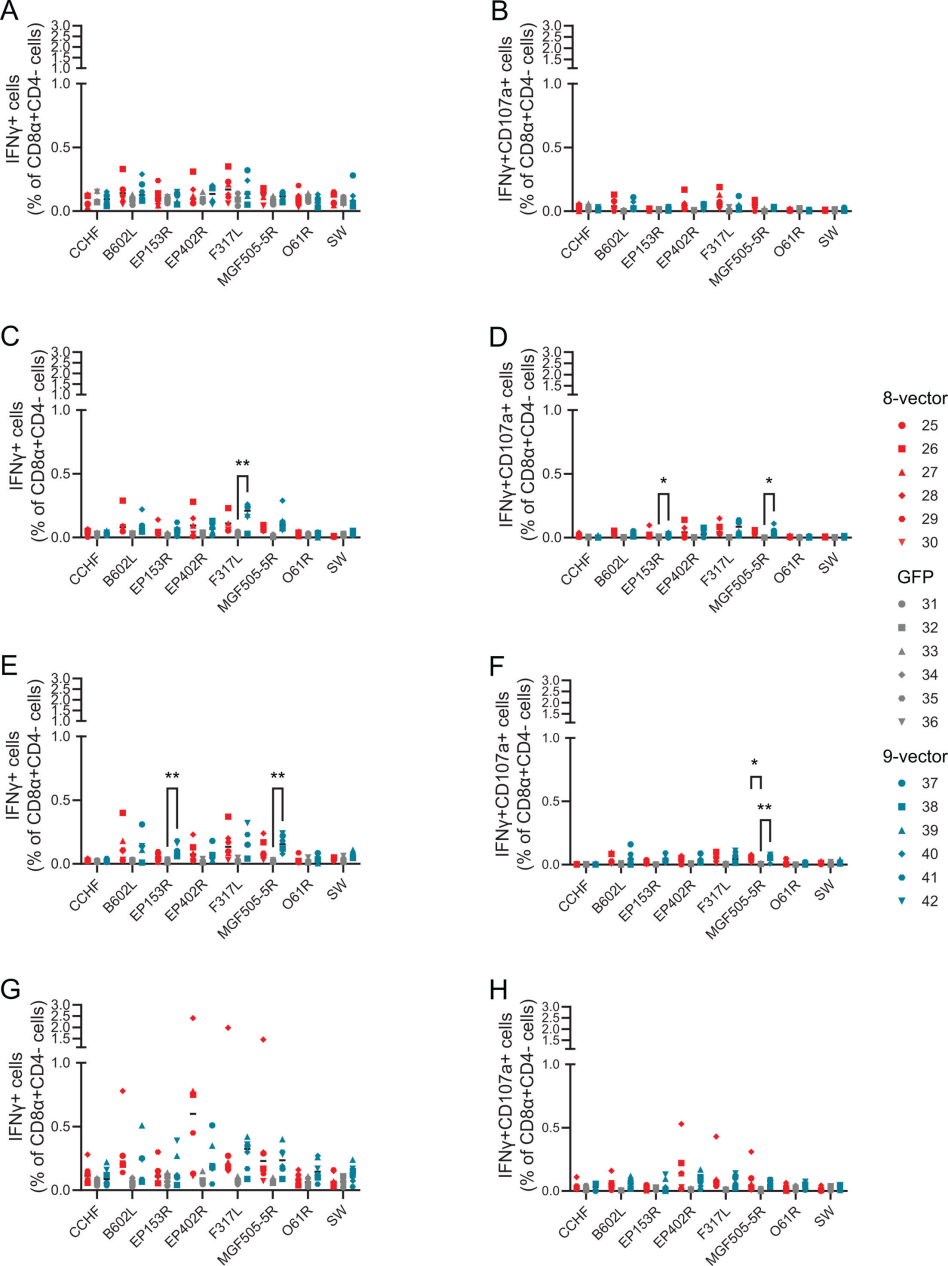
Antigen-specific CD8 T-cell responses. Animals were immunized with eight vectors (red), GFP (gray), or nine vectors (blue) and boosted 28 days later. PBMCs purified on the indicated days were stimulated with peptide pools corresponding to the indicated gene products or the ubiquitinylated polyprotein (SW). The proportion of CD3^+^CD8α^+^CD4 cells expressing IFNγ (**A–D**) or IFNγ and CD107a (**E–H**) 2 weeks post-prime (**A, D**), pre-boost (**B, E**), 7 days post-boost (**C, F**), and pre-challenge (**D, H**) was determined by flow cytometry. Each data point indicates a single animal, and bars show the mean of each group. Green fluorescent protein (GFP); interferon gamma (IFNγ); peripheral blood mononuclear cell (PBMC). *P ≤ 0.05, ** ≤ 0.01*.*

Unfortunately, the CD3 staining failed on the first day of the experiment, and therefore, we cannot rule out the possibility that antigen and virus responses reported after that point were not specific. However, we judge this unlikely as we have never seen significant background responses after restimulation with whole ASFV or peptide pools in previous adenovirus studies in pigs (10, 11, 13, 62). Antigen-specific CD4 (Fig. 1) and CD8 (Fig. 2; Fig. S4) T-cell responses could be detected in individual pigs 2 weeks post-prime. CD4 responses to pB602L and pF317L reached significance at the group level 2 weeks post-prime (Fig. 1A), and CD8 responses to pF317L were significant 4 weeks post-prime (Fig. 2C). Cell surface expression of CD107a by CD8 after stimulation with ASFV antigens could be detected in individual pigs as early as 14 days post-prime (Fig. 2B), and at the group level to pEP153R and pMGF505-5R 4 weeks post-prime (Fig. 2D), suggesting that the immunizations had induced antigen-specific CD8 T cells with potential cytolytic activity. Numbers of antigen-specific multi-functional CD8 T cells that also expressed TNF as well as IFNγ and CD107a were relatively low (Fig. S4B, D, F and H), but pEP402R and pMGF505-5R specific cells could be detected 4 weeks post-prime (Fig. S4D). pO61R antigen-specific responses were not detected in any pigs at any time points. Responses to the ubiquitinylated polyprotein were assessed using peptides to pI73R and pCP204L, and these were detected in only two cases, CD8 cells from pig 38 on Day 14 (Fig. 2A) and CD4 cells from pig 41 on Day 35 (Fig. 1C). Significant differences in the CD4 or CD8 responses between the control group and the eight-vector and nine-vector groups could be detected against the other antigens on at least one of the measured time points. Although antigen-specific responses could be detected in individual animals in both groups immunized with ChAdOx1 vectors expressing ASFV antigens, significant differences to the controls were generally only seen in the nine-vector group. The percentage of antigen-specific cells did not increase after the boost (compare Day 28 data in Fig. 1 and 2 and Fig. S4) and prior to challenge with virulent ASFV on Day 52, there were no significant differences for any of the antigen-specific responses when either the eight-vector or nine-vector group was compared to the controls (Day 52 data in Fig. 1 and 2; Fig. S4).

**Fig 3:**
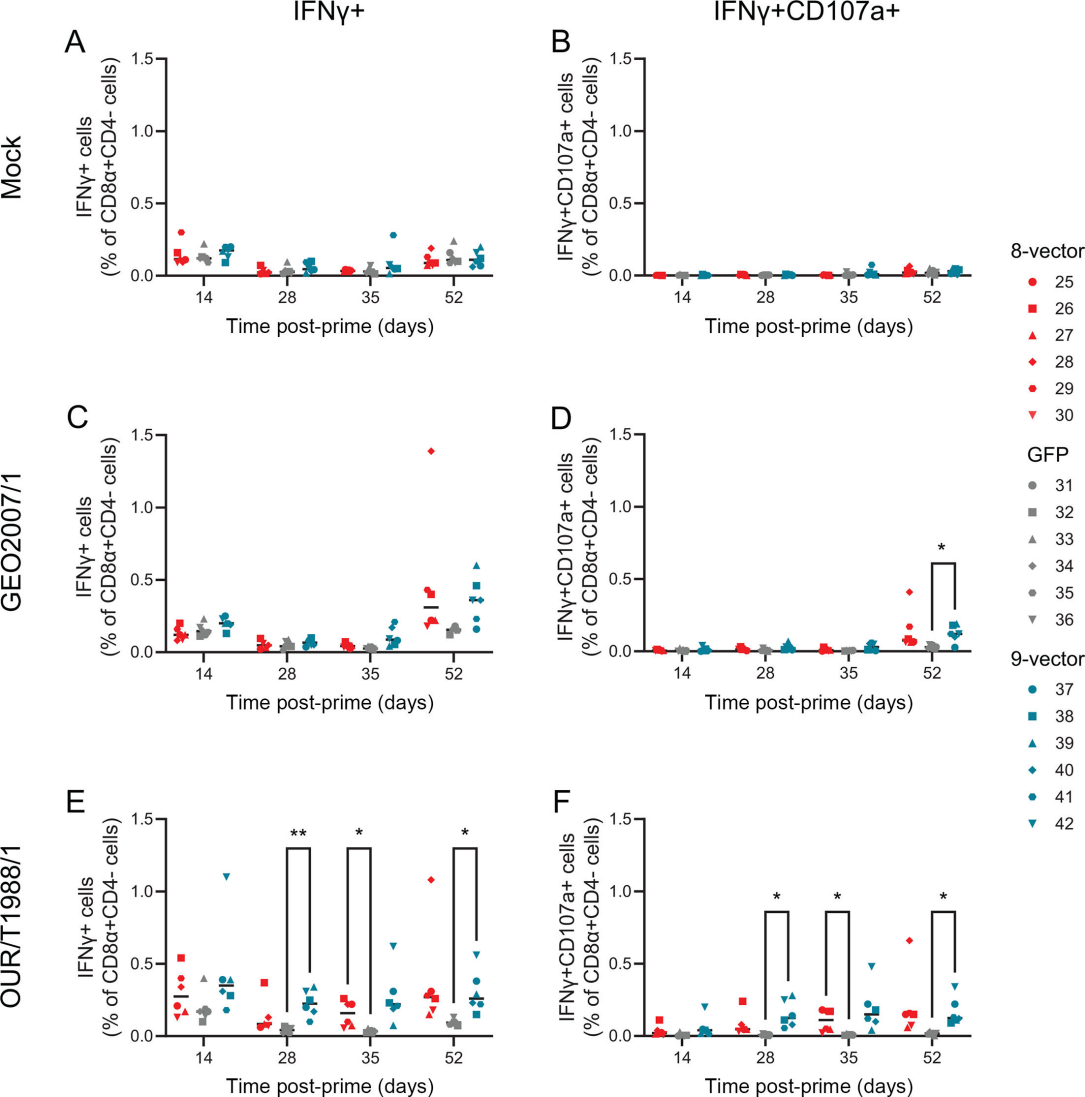
Virus-specific CD8 T-cell responses. Animals were immunized with eight vectors (red), GFP (gray), or nine vectors (blue). Cells were purified from blood collected on the indicated days and stimulated with mock (**A, B**), OUR T1988/1 (**C, D**), or Georgia 2007/1 (**E, F**) inocula overnight and then treated with brefeldin A and anti-CD107a for 4 h. Populations of CD3^+^CD8α^+^CD4 cells were then identified by flow cytometry, and the proportion of them expressing either IFNγ (**A, C, E**), IFNγ and CD107a (**B, D, F**) was determined. Each data point indicates a single animal, and bars show the mean of each group. Green fluorescent protein (GFP); interferon gamma (IFNγ). *P ≤ 0.05, ** ≤ 0.01*.*

Weak virus-specific CD8 (Fig. 3; Fig. S5) and CD4 (Fig. S6 and S7) responses were found at all time points measured; however, these were primarily after stimulation with the genotype I OUR T1988/1 isolate rather than genotype II virus that the protein sequence of the antigens was derived from. Significant differences in the percentage of Georgia 2007/1 multifunctional CD8^+^CD4 cells (Fig. 3D; Fig. S5C and D) and CD4^+^CD8^+^ cells (Fig. S6C, D and S7C) were observed, between the controls and the other groups; however, both the percentages of cells and numbers of cytokine-producing CD8 cells (Table S1) were very low for all but CD8^+^CD4^−^IFNγ^+^ cells (Fig. 3E). No differences were observed between the responses seen in the animals given eight vectors and those given nine vectors for any of the cell types measured; however, statistical significance between the GFP control group and the nine-vector group was more commonly seen than between the GFP control group and the eight-vector group (Fig. 3E and F; Fig. S5E and F). CD8^+^CD4^−^ responses after Georgia 2007/1 restimulation of peripheral blood mononuclear cells (PBMCs) collected at pre-challenge were observed to be at best double that of the control group with the exception of Pig 28 from the eight-vector group (Fig. 3C and D; Fig. S5C and D). Taken together, this suggested that the ChAdOx1 vectors induced weak antigen-specific responses and that these were associated with poor virus-specific responses, particularly against genotype (II Georgia 2007/1).

**Fig 4:**
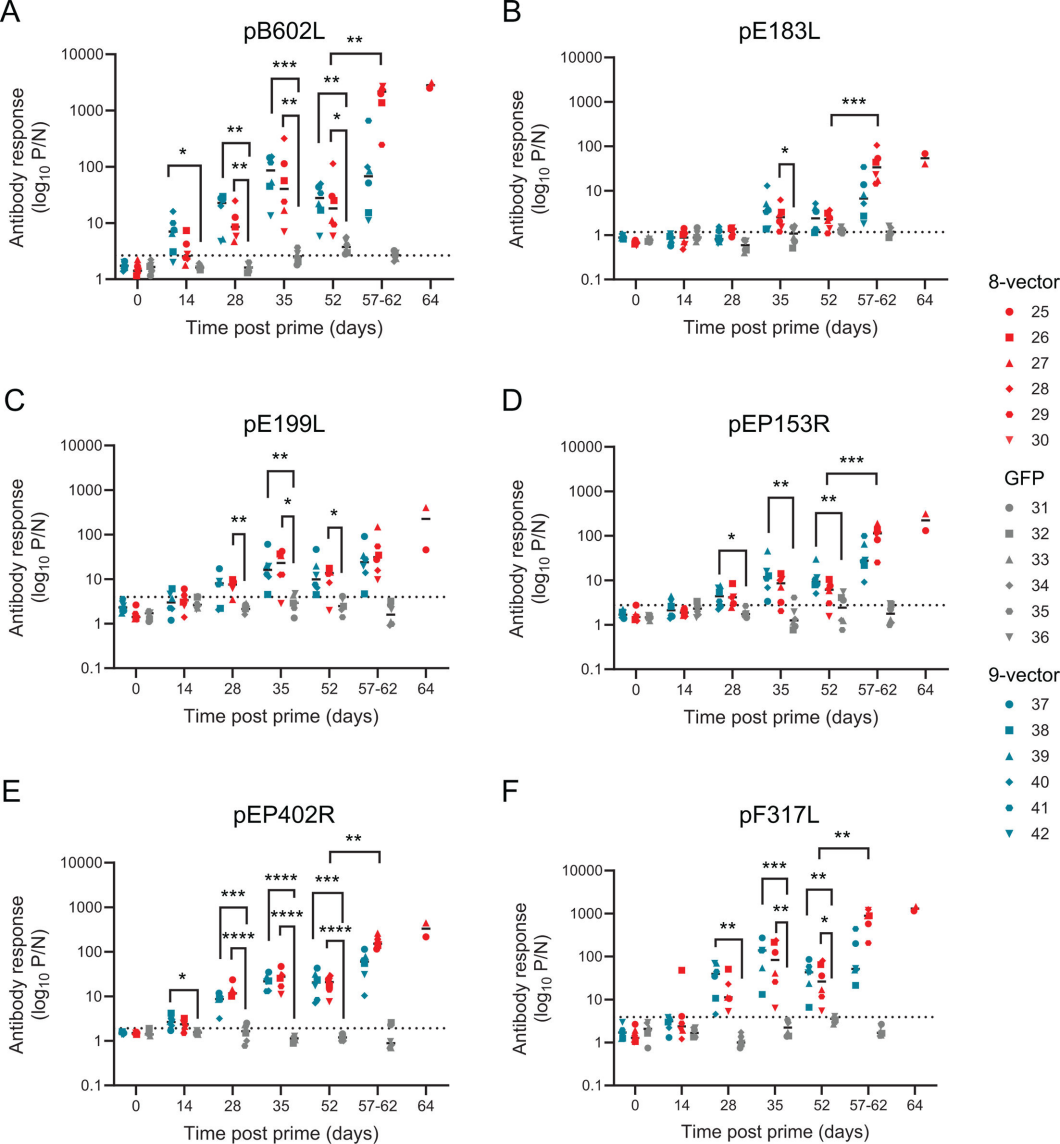
Antigen-specific antibody responses. Animals were immunized with eight vectors (red), GFP (gray), or nine vectors (blue), boosted 28 days later, and then challenged on Day 52 (V). Serum was collected on the indicated days, and antibody responses against pB602L (**A**), pE183L (**B**), pE199L (**C**), pEP153R (**D**), pEP402R (**E**), and pF317L (**F**) were determined by LACA. The negative cutoff for each protein-specific LACA was determined from the mean and 3× standard deviation of all negative sera samples in each experiment and is denoted by a dashed line in each graph. Only time points between Day 0 and Day 52 were included in statistical analyses. Each data point indicates a single animal, and bars show the mean of each group. Green fluorescent protein (GFP); Luciferase-linked antibody capture assay (LACA). *P ≤ 0.05, ** ≤ 0.01, ***P ≤ 0.001, and ****P ≤ 0.0001.

**Fig 5:**
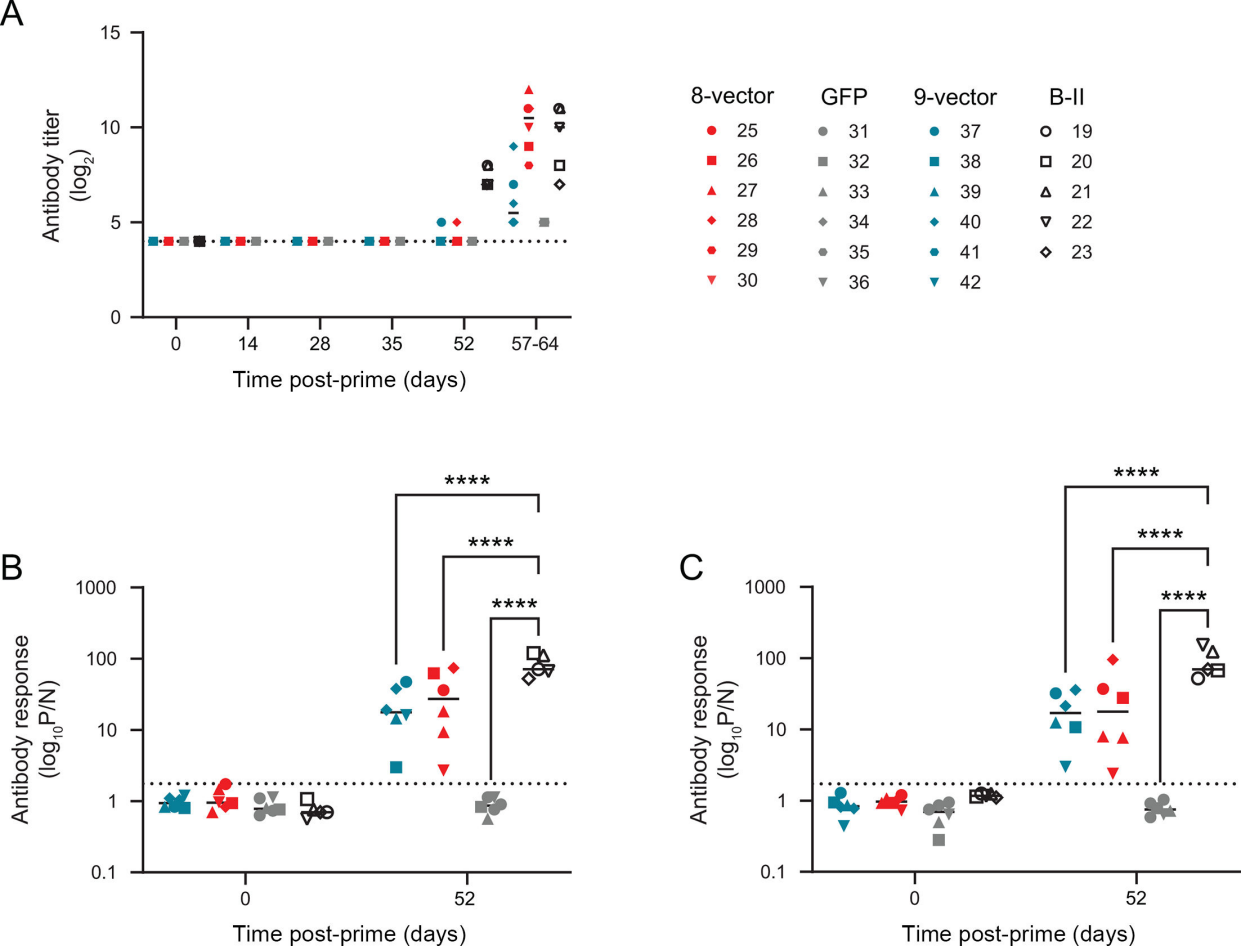
Virus-specific antibody responses. (**A**) Animals were immunized with eight vectors (red), GFP (gray), or nine vectors (blue), boosted 28 days later, and then challenged on Day 52. Serum was collected on the indicated days, and antibody responses against ASFV-infected cells were determined by immunoperoxidase assay. Open black symbols show equivalent results from Group B-II immunized with HuAd5 vectors expressing both genotype I and genotype II antigens. Comparison of B602L (**B**) and F317L (**C**) antibody responses as measured by LACA between the present experiment and a genotype I/II vaccine. Each data point indicates a single animal, bars show the mean of each group, and the dotted line indicates the limit of detection of the assay. African swine fever virus (ASFV); green fluorescent protein (GFP); Luciferase-linked antibody capture assay (LACA). ****P ≤ 0.0001.

Antibody responses against pB602L, pE183L, pE199L, pEP153R, pEP402R, and pF317L were determined using luciferase-linked antibody capture assays (Fig. 4). We were unable to reliably detect antibody responses to either pO61R or pMGF505-5R, and sera from pigs recovered from genotype II ASFV did not cross-react with cells transiently expressing pMGF505-5R (not shown), suggesting robust antibody responses to at least pMGF505-5R were not induced in either group. It is possible that antibodies to pO61R were produced, but due to the lack of a reliable assay, these were not measured. Antibody responses to pB602L could be detected as early as 14 days post-prime and to the other antigens from 4 weeks post-prime. Antibody responses increased immediately after the boost, but statistically significant differences between antibody responses pre-boost (Day 28) and pre-challenge (Day 52) were only found for pE183L (Fig. 4B) and pEP153R (Fig. 4D). No antibody responses were detected using an anti-p30 competitive ELISA (Fig. S8), p30 was a component of the ubiquitinylated polyprotein and one of the most immunogenic ASFV proteins. Despite the antigen-specific responses, no virus-specific antibody responses were observed in any animals prior to challenge (Fig. 5A) despite a combination of HuAd5 vectored genotype II *B602L*, *E183L*, *EP153R* and genotype I *E199L*, *F317L*, and *MGF505-5R* inducing virus-specific antibody responses in Group B-II in a previous experiment (13). Analysis of the pre-challenge responses to pB602L (Fig. 5B) and pF317L (Fig. 5C) was higher in group B-II from this previous study, suggesting that lower antigen-specific responses were leading to poorer virus-specific responses. Virus-specific antibody responses were in sera collected at termination; however, as these were collected 5–12 days post-challenge, these could be primary responses to the virus challenge, rather than secondary responses.

**Fig 6:**
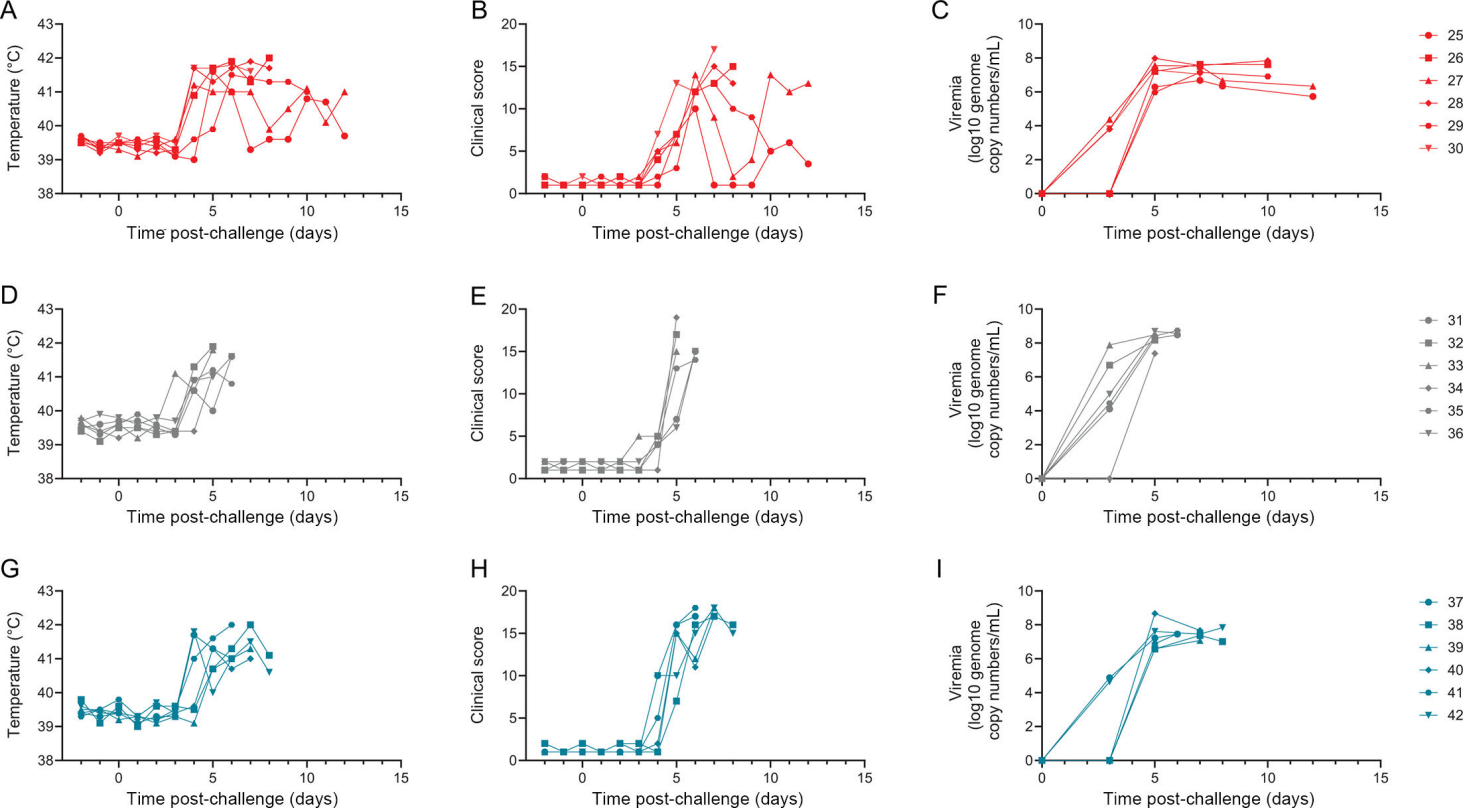
Clinical and virological data for individual animals. Pigs immunized with eight vectors (red, **A, D, G**), GFP (gray, **B, E, H**), or nine vectors (blue, **C, F, I**) were challenged with ASFV Georgia 2007/1 (Day 0). Rectal temperatures (**A–C**) and clinical scores (**D–F**) were measured daily, and blood samples were collected on the indicated days to measure viremia by qPCR (**G–I**). Each data point indicates a single animal. African swine fever virus (ASFV); Green fluorescent protein (GFP).

To assess the protective efficacy of the pools of antigens, the animals were challenged via the intramuscular route with Georgia 2007/1 4 weeks after the boost (Day 52). Clinical signs were first observed in the control group with pig 32 showing a temperature of 41.1°C 3 days post-challenge (Fig. 6A through C), other clinical signs such as inappetence and recumbency were observed from 4 days post-challenge onwards (Fig. 6D through F). The control pigs were culled 5 or 6 days post-challenge, and the pigs immunized with nine vectors were culled 8 or 9 days after infection. Different patterns were observed in the group of pigs immunized with the eight vectors after challenge. Pigs 26, 28, and 30 followed a similar pattern to the pigs immunized with nine vectors with persistent high fever and were culled 7 and 9 days post-challenge (Fig. 6A and D). Pigs 25 and 27 showed a transient fever of 1–3 days, then showed signs of recovering before fever re-emerged; both animals were culled 12 days after infection. Pig 29 demonstrated a prolonged fever and was culled 10 days post-challenge. Ultimately, neither combination of vectors protected the animals from severe disease caused by ASFV. Macroscopic lesions typically seen in pigs suffering acute ASF were observed at postmortem in all of the animals (Fig. S9A and B) and virus was identified in all tissues tested, with a lower viral load generally seen in the nine-vector group (Fig. S9C). Taken together, the data demonstrated a minor reduction in viral load in some tissues, but this was insufficient to protect the animals from clinical disease.

## DISCUSSION

An African swine fever subunit vaccine would help alleviate concerns around the safety of modified live virus vaccines including those currently deployed in Vietnam. However, results in challenge experiments with the predominant circulating virulent genotype II isolate have to date not been successful. In our previous study, HuAd5 vectored genotype I *E199L*, *F317L*, and *MGF505-5R* in combination with genotype II *B602L*, *E183L*, and *EP153R* did not prevent acute disease after intramuscular challenge with the prototype genotype II virus Georgia 2007/1 (13). One possible explanation for the failure to protect against genotype II ASFV was the amino acid differences between the genotype I proteins used in this experiment and those present in the challenge virus. However, the differences between the protein sequences of genotype I and genotype II pE199L, pF317L, and pMGF505-5R are relatively minor, being three, one, and eight amino acids, respectively. As such, it is unlikely that these differences alone were the reason for success against genotype I and failure with genotype II. Two additional vectors expressing *EP402R* and *O61R*, as well as a ubiquitinylated polyprotein containing several potential T-cell antigens, were included to try and increase the chances of delivering protective immune responses.

Unfortunately, neither immunization with ChAdOx1 expressing *B602L*, *E183L*, *E199L*, *EP153R*, *EP402R*, *F317L*, *MGF505-5R*, and *O61R*, nor immunization with the same eight vectors in combination with a ChAdOx1 vector expressing *I73R*, *F334L*, *CP204L*, *M448R*, and *B646L* genes in frame with ubiquitin prevented acute disease after challenge with virulent genotype II ASFV. Clinical progression after ASFV challenge was generally similar to that previously observed with the combination genotype I/II vaccine (13). Some pigs had a more prolonged course of disease, whereas others suffered clinical signs consistent with acute ASF. However, in our previous study, the combination genotype I/II vaccine caused a short delay in the appearance of both clinical signs and viremia, and this did not occur in the present study. Taken together, the data presented show that the two pools of replication-deficient simian adenoviruses did not prevent development of clinical signs consistent with acute ASF. We also cannot rule out that these animals would have recovered if we had not allowed the disease to take its natural course.

Antigen-specific antibody or cellular immune responses were detected to all of the antigens apart from pO61R in at least one pig. The responses to the ubiquitinylated polyprotein were also poor with a positive response only seen in two animals, one pig 2 weeks post-prime and another pig 1 week post-boost. However, it is important to note that only peptides corresponding to pI73R and pCP204L were used to assess the response to the polyprotein, and so we cannot rule out that responses to pB646L, pF334L, or pM448R were present but not detected. Fusion of ubiquitin to the N-terminus of the polyprotein was intended to drive immune responses toward cellular immunity and away from humoral immune responses, as has been successfully employed in DNA vaccination studies (59, 68). Consistent with this, we were unable to detect antibody responses to the highly immunogenic pCP204L/p30 (10, 62, 69); however, we once again cannot rule out antibody responses to other parts of the polyprotein, as these were not tested.

Georgia 2007/1 specific CD8 responses were also poor with the exception of pig 28 in the eight-vector group. Consistent with the higher CD8 responses to virus, pig 28 also had higher pre-challenge responses to a number of the antigens (Fig. 3G); however, this animal had worse disease outcome than pigs 25 and 27, showing that elevated virus-specific cellular immune responses did not lead to an improved clinical picture. Antigen-specific CD4 responses were observed in a number of animals, and although virus-specific IFNγ^+^ cells were identified, the numbers of events detected were very low. Due to the failure of the CD3 labeling of the pre-prime flow cytometry, we cannot rule out that the antigen-specific and virus-specific responses we observed in the eight-vector and nine-vector groups were non-specific; however, the absence of responses in the control group leads us to conclude that this is unlikely to be the case.

As previously shown, cellular immune responses from T cells after restimulation with genotype I PRT/OUR/T1988/1 were better than those after the challenge virus, Georgia 2007/1 (13). Georgia 2007/1 does not block T-cell responses in restimulation assays with cells from pigs immunized with MLVs (70–72) and therefore it seems likely that there are fundamental differences in the nature of the cellular immune response elicited by MLVs compared to those that are induced by replication-deficient adenoviruses. A detailed comparative analysis of genotype I rAd induced cellular immune responses with those induced by both genotype I and II MLVs may yield further insights that could be exploited in future vaccine designs. It is also possible that the SLA haplotype of the animals influenced the cellular immune response to individual antigens and the virus. Although our previous analysis did not identify a relationship between SLA and immunological or clinical outcomes (10, 62), a wider analysis of antigen-specific responses and SLA haplotypes in this and other studies may clarify this point.

The absence of virus-specific antibody responses was surprising and likely linked to the apparent reduced antigen-specific responses in comparison to our previous study with HuAd5 vectors. Although we do not have directly comparable studies in terms of vectors, doses, and antigens, all of our previous HuAd5 experiments that included a subset of these antigens induced ASFV-specific antibody responses (10, 13). Four bivalent ChAdOx1 expressing a set of ASFV genes unrelated to the present study were able to induce antibodies capable of recognizing ASFV-infected cells (11), and a monovalent ChAdOx1 vector expressing SARS-CoV-2 spike protein induced SARS-CoV-2 neutralizing antibodies in pigs (73). Differences in the immunogenicity of different replication-deficient adenoviruses have been reported (74–78), with transgene expression driven from ChAdOx1 performing more poorly in draining lymph nodes compared to HuAd5 in mouse models suggesting this vector may be more efficient at entering antigen-presenting cells (76). However, comparative data in pigs with both single vectors and complex multi-vector pools are needed to draw meaningful conclusions on the relative performance of different adenovirus platforms with regard to both antigenicity and antigen competition.

HuAd5 and ChAdOx1 are human adenovirus species C (*Mastadenovirus caesari*) and E (*Mastadenovirus exoticum*), respectively, and use the coxsackie and adenovirus receptor (CAR) as an entry receptor (79–81). Swapping the receptor binding domain between ChAdOx1 and HuAd5 led to altered host range *in vitro,* and there was no effect on immunogenicity *in vivo* (76). Other adenovirus species can use different molecules as receptors, including CD46 (82). Receptor binding and cell tropism data for adenoviruses in pig cells are not available, and although porcine and human CAR are 90% identical, it is possible that HuAd5 and ChAdOx1 may have different binding affinities for pig CAR, or that HuAd5 and ChAdOx1 could enter different cells in pigs.

Interestingly, a combination of *B602L*, *B646L*, *CP204L*, *E183L*, and *EP402R* vectored using HuAd2 (*M. caesari*) protected pigs against ASFV in a farming setting (65). The authors of this paper evaluated a number of human adenovirus vectors and selected HuAd2 based on levels of transgene expression in porcine kidney cells. It will be interesting to see if *B602L, B646L*, *CP204L*, *E183L*, and *EP402R* vectored by HuAd2 or another vaccine platform can protect animals from ASF in direct challenge studies with both genotype II and genotype I/II hybrid viruses.

## MATERIALS AND METHODS

### Cells and viruses

African swine fever virus isolates PRT/OUR/T1988/1 (PX548505) and Georgia 2007/1 (FR682468.2) have been described previously (83, 84) and were grown and titrated in porcine bone marrow-derived macrophage cultures (85). The Vero cell-adapted Badajoz 1971 strain (Ba71v) has been described previously (86) and was cultured and titrated on Vero cells, which were maintained in DMEM-HEPES supplemented with 10% heat-inactivated fetal calf serum and 100 IU/mL penicillin and 100 µg/mL streptomycin. HEK293T cells were maintained using the same conditions as Vero cells.

Peripheral blood mononuclear cells (PBMCs) were purified from heparinized blood using Histopaque and then cultured in RPMI GlutaMax supplemented with 25 mM 4-(2-hydroxyethyl)-1-piperazineethanesulfonic acid, 50 µM 2-mercaptoethanol, 10% (vol/vol) fetal calf serum, 100 units/mL penicillin, 100 µg/mL streptomycin, and 1 mM pyruvate (RPMI/10).

### Viral vectors

Production of clonal ChAdOx1 vectors is described in detail in supplementary methods. Briefly, codon-optimized genes were designed using amino acid sequences obtained from the Georgia 2007/1 isolate of ASFV (FR682468.2). The fusion protein was comprised of a 5′ to 3′ fusion of *Sus scrofa* ubiquitin C gene with the ASFV genes *I73R*, *F334L*, *CP204L*, *M448R*, *B646L* with the sequence of a V5 epitope tag at the 3′ end in a single open reading frame. ChAdOx1 vectors were produced in T-REx-293 cells (ThermoFisher Scientific). ChAdOx1-GFP has been described previously (87).

### Animal studies

The animals were housed in accordance with the Code of Practice for the Housing and Care of Animals Bred, Supplied or Used for Scientific Purposes, and bedding and species-specific enrichment were provided throughout the study to ensure high standards of welfare. Through careful monitoring, pigs that reached the scientific or humane endpoints of the studies were euthanized by an overdose of anesthetic. All procedures were conducted by Personal License holders under the auspices of Project License PP8739708.

Eight-week-old (20 kg) female Landrace × Large White × Hampshire pigs were obtained from a high health farm in the United Kingdom and randomly assigned to each group prior to immunization. Piglets had been vaccinated against porcine circovirus type 2 (subunit) and sows and gilts were vaccinated against *Escherichia coli* (multiple subunits of adhesins and a toxin), Erysipelas (inactivated), and Parvovirus (inactivated). The farm has been free of porcine reproductive and respiratory syndrome virus since at least 2019. No baseline immune measurements were taken before the start of the study; however, all pigs were clinically normal on arrival and were acclimatized for 7 days before the start of the experiment. Pigs were randomly assigned to groups on arrival.

Groups of six pigs were inoculated intramuscularly in the neck with either eight rAd-expressing individual ASFV ORFs (Pool A, pigs 25–30), eight rAd-expressing individual ASFV ORFs as well as an rAd expressing the ubiquitinylated fusion protein (Pool B, pigs 31–36), or with rAd-GFP (Pool C, pigs 37–42). Each rAd was administered at a dose of 5 × 10^9^ infectious units (IU), therefore all animals were primed with a maximum dose of 4.5 × 10^10^ IU. Twenty-eight days later, animals were boosted with the same combinations of vectors at the same site. Blood samples were collected before each immunization (0 and 28 days post-prime) and 2 weeks after prime (14 days post-prime), and 1 week after boost (35 days post-prime). Pigs were then moved to the SAPO4 high containment laboratories at the Pirbright Institute for ASFV challenge, and blood samples were taken immediately before challenge (52 days post-prime) and at regular intervals afterward (Fig. S3). Pigs were challenged with 1 × 10^3^ hemadsorbing units of Georgia 2007/1 by the intramuscular route in the neck. Macroscopic lesions identified at post-mortem were scored using standard scoring methodology (88, 89).

Group B-II has been described previously (13). Briefly, five female Landrace × Large White × Hampshire pigs were primed and boosted 4 weeks apart with human adenovirus five vectors expressing genotype I *E199L*, *F317L*, and *MGF505-5R* and genotype II *B602L*, *E183L,* and *EP153R*. Each vector was used at a dose of 1.5 × 10^10^ IU.

### Quantitative real-time PCR

The previously published assay to determine ASFV genome copies in whole blood and tissue samples was used with slight modifications (90). Briefly, 20 mg of tissue was homogenized with RPMI in a Lysing Matrix A 2 mL tube (MP Biomedicals) using the BeadBug homogenizer. Nucleic acid was extracted from homogenized tissue or blood samples with the MagMAX Core nucleic acid extraction kit (Thermo Fisher Scientific) and KingFisher Flex (Thermo Fisher Scientific) according to the manufacturer’s instructions. Primers Fwd 5′-CTGCTCATGGTATCAATCTTATCGA-3′, Rev 5′-GATACCACAAGATCRGCCGT-3′ and the probe 5′-(6-carboxyfluorescein[FAM])-CCACGGGAGGAATACCAACCCAGTG-3′-(6-carboxytetramethylrhodamine [TAMRA] were used in qPCRs performed on the Quantstudio 5 system (Thermo Fisher Scientific) with a two-step thermal profile: 95°C for 10 min and 45 cycles of 95°C for 15 s and 60°C for 60 s. No template controls and plasmid standards were included on all plates.

### Flow cytometry

Peripheral blood mononuclear cells (PBMCs) were isolated from heparinized blood samples, seeded at 1.5 × 10^6^ cells per well, and incubated overnight with live virus (Georgia2007/1 and PRT/OUR/T1988/1, MOI = 0.5 HAD_50_), mock inoculum, or pools of peptides. Peptides supplied in 50% acetonitrile/50% water (vol/vol), 0.1% trifluoroacetate (Thermo Scientific) were used, and the maximum number of peptides used in any one pool was 65. Optimal concentration of peptide pools was titrated using cryopreserved PBMCs from convalescent animals and the median concentration of each individual peptide incubated with cells was 1.45 µg/mL. For pB602L, pEP153R, pEP402R, pF317L, pMGF505-5R, and pO61R, 16-mer peptides overlapping by eight amino acids were used. 20-mer peptides overlapping by 10 amino acids for pI73R and pCP204R were used to represent the ubiquitinylated fusion protein and five 20-mer peptides encoding the Gc protein of Crimean-Congo hemorrhagic fever virus were used as the negative control as previously described (11). All peptides for ASFV proteins were derived from the Georgia 2007/1 sequence (Table S1). Cells were treated with 5 μg/mL Brefeldin A (BioLegend) the next morning and positive controls were stimulated with 100 ng/mL phorbol 12-myristate 13-acetate (Merck) and 2 µg/mL ionomycin (Merck) for another 4 h. For flow cytometry analysis, cells were stained using the antibodies and dilutions outlined in Table 1. The fixable live/dead stain Zombie NIR (1:500 dilution, BioLegend) was used to identify live cells. Surface staining was performed at room temperature for 15 min before fixation and permeabilization with BD Cytofix/Cytoperm (BD Biosciences) for 30 min in the dark at room temperature. For intracellular staining, directly conjugated antibodies were first incubated for 15 min at room temperature, before two washes and final staining with streptavidin-BV650 for 15 min at room temperature. Cells were acquired on a LSR Fortessa (BD Biosciences) and analyzed using FlowJo 10. Percentages of individual cell populations, as well as the number of events, are reported in Table S1. A mean of 14.5% and 27.2% of PMA-treated CD3^+^CD4^+^CD8α^+^ cells expressed IFNγ or TNF, respectively, and 7.7% and 13.3% of PMA-treated CD3^+^CD8α^+^CD4 cells expressed IFNγ or TNF, respectively (Table S1).

**TABLE 1:** Antibodies used for flow cytometry staining

Antigen	Clone	Isotype	Conjugate	Dilution used	Source of Ab	Cat. no.
Extracellular						
CD3	BB23-8E6-8C8	Mouse IgG2a	PE-Cy7	1:50	BD Biosciences	561477
CD4	74-12-4	Mouse IgG2b	PerCP-Cy5.5	1:50	BD Biosciences	561474
CD8α	MIL12	Mouse IgG2a	FITC	1:50	Bio-Rad Laboratories	MCA1223F
CD107a	4E9/11	Mouse IgG1	Alexa Fluor 647	1:100	Bio-Rad Laboratories	MCA2315A647
Intracellular						
IFNγ	P2G10	Mouse IgG1	PE	1:100	BD Biosciences	559812
TNF	MAb11	Mouse IgG1	BV421	1:50	BioLegend	502932
IL-2-biotin	A150D 8H10	Mouse IgG1	Biotin	1:400	Invitrogen	ASC0829
Biotin	NA^*a*^	NA	Streptavidin-BV650	1:500	BioLegend	405231

^*a*^ NA, not applicable.

The gating strategy is shown in Fig. S10 and fluorescence minus ones in Fig. S11. Unfortunately, the CD3 labeling for the pre-prime (Day 0) samples failed and therefore these data are missing.

### Luciferase-linked antibody capture assays (LACAs)

A more detailed method to generating a modified LACA for the characterization of longitudinal ASFV antigen-specific antibody responses has previously been published (91). Briefly, plasmids expressing Nluc-tagged recombinant antigens B602L, E183L, E199L, EP153R, EP402R, and F317L were first generated using pig codon optimized sequences derived from ASFV Georgia2007/1 (GenBank accession no. NC_044959). HEK293T cells were then transfected with these plasmids using TransIT-LT1 (Mirius Bio) to obtain antigen lysates with the *Renilla* Luciferase Assay (RLA) kit (Promega) for use in LACAs.

All incubations were performed at room temperature unless indicated. Multi-well LumiNunc opaque white plates (Thermo Scientific) that were coated overnight with 8 µg/mL Protein A from *Staphylococcus aureus* (Merck) in Carbonate-Bicarbonate buffer (Merck) at 4°C were first washed three times with wash buffer (0.5% (vol/vol) Triton-X100 (Merck) in 1× PBS). Plates were subsequently blocked with blocking buffer (5% skim milk in wash buffer) for 2 h. In parallel, antigen lysates (diluted to 2 × 10^7^ ALU/mL in block buffer) and sera samples (diluted 1:50 in block buffer) were mixed at a ratio of 1:1 and incubated for 1 h with agitation. Sera-antigen lysate mixes were then transferred into the blocked plates and incubated for 1 h with agitation. After six washes with wash buffer and two washes with 1× PBS, luciferase activity was measured with RLA and a Cytation 3 multi-mode reader (Biotek). Data are expressed as the ratio of luciferase activity in each sample to that of the negative control, FCS (P/N ratio). Cutoff values for each antigen were determined by the mean plus 3× standard deviation of the P/N ratios from all negative sera in each experiment. Sera collected 59 days after immunization from animal K5 (72) was used as a positive control and achieved P/N values of 346.10, 10.75, 31.65, 2.35, 67.83, and 11.54 for pB602L, pE183L, pE199L, pEP153R, pEP402R, and pF317L, respectively.

### Fixed-cell ELISA

Vero cells 16 hpi with Ba71v were fixed with 4% paraformaldehyde, permeabilized, and then labeled with twofold serial dilutions of sera as described previously (13). Sera collected 63 days after immunization from animal AV73 (54) was used as a positive control and achieved a titer of ≥13 log_2_.

### Statistics

Statistical analyses were performed using GraphPad Prism (v10.4, GraphPad Software, LLC). Differences between groups were identified using repeated measures mixed-effects model (RM-REML) with the Geisser-Greenhouse correction. Multiple comparisons were performed using Tukey’s multiple comparisons test, with individual variances computed for each comparison. Differences in immune responses are only reported if differences were also identified between a given treatment and a control treatment within a group (RM-REML *P* ≤ 0.05). For example, differences between the antibody responses to pEP402R between the control and eight-vector group (Fig. 4E) are reported because there was also a difference between the antibody responses in the eight-vector group on Day 52 compared to Day 0. *P* values and confidence intervals are reported in Table S1 and *P* values in the figures are reported as; *P ≤ 0.05, ** ≤ 0.01, ***P ≤ 0.001, and ****P ≤ 0.0001.

## Data Availability

All raw data are included in the text, figures, or in Table S1.

## SUPPLEMENTAL MATERIAL

Data S1 spectrum.02328-25-s0001.xlsx Raw data, antigen, and peptide sequences.

Fig. S1 spectrum.02328-25-s0002.tif Confocal images.

Fig. S2 spectrum.02328-25-s0003.tif Light microscopy images.

Supplemental material spectrum.02328-25-s0004.pdf Supplemental methods; Fig. S3 to S11.

ASM does not own the copyrights to Supplemental Material that may be linked to, or accessed through, an article. The authors have granted ASM a non-exclusive, world-wide license to publish the Supplemental Material files. Please contact the corresponding author directly for reuse.
