# Supplementary figures and images for: African swine fever virus genes vectored by simian adenoviruses do not protect against virulent genotype II virus challenge
Source: Microbiol Spectr. 2026 Feb 27;14(4):e02328-25. doi: 10.1128/spectrum.02328-25 (PMC13055240; doi:10.1128/spectrum.02328-25)

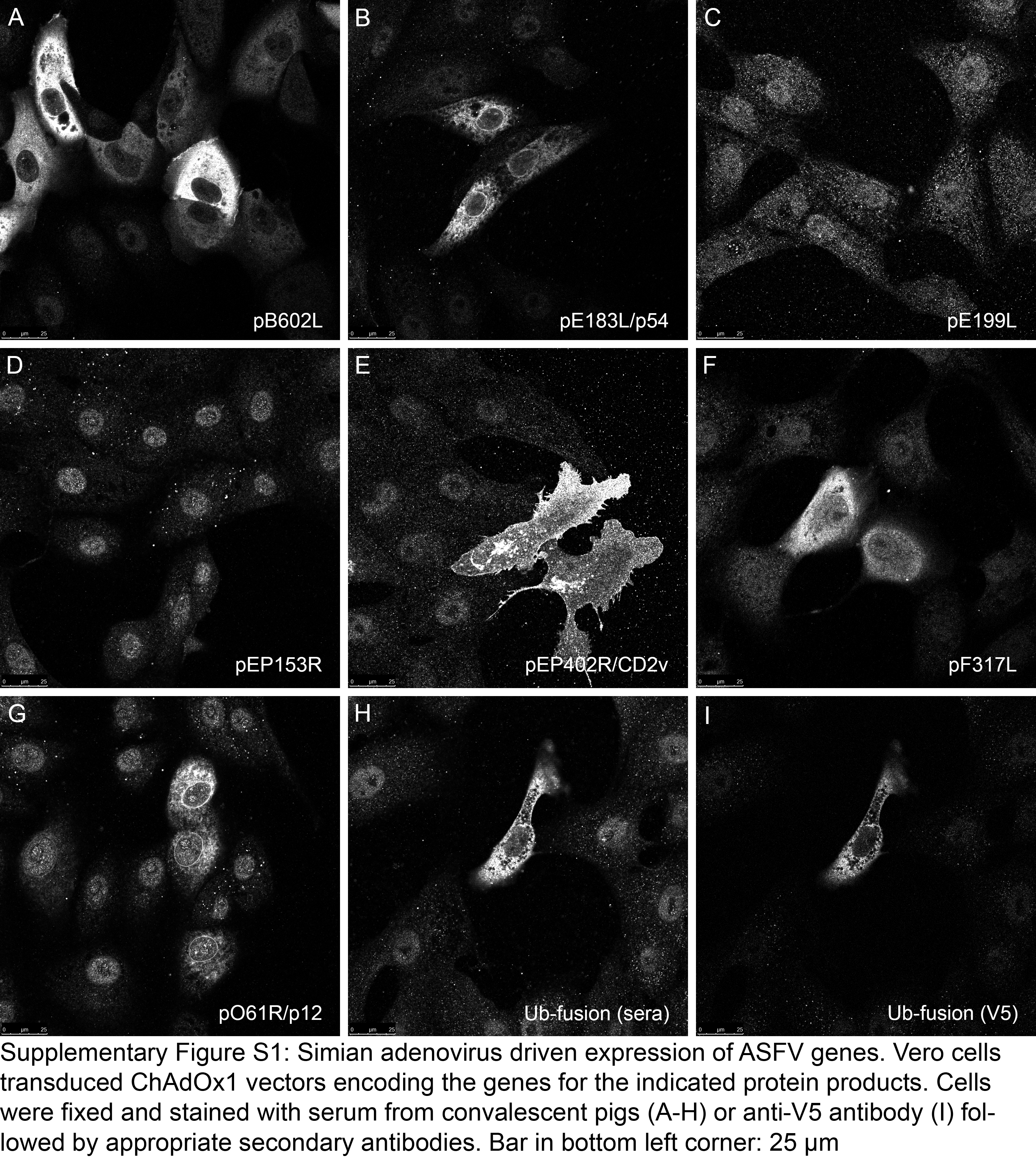

Supplement: Fig. S1 — Confocal images. [file spectrum.02328-25-s0002.tif]

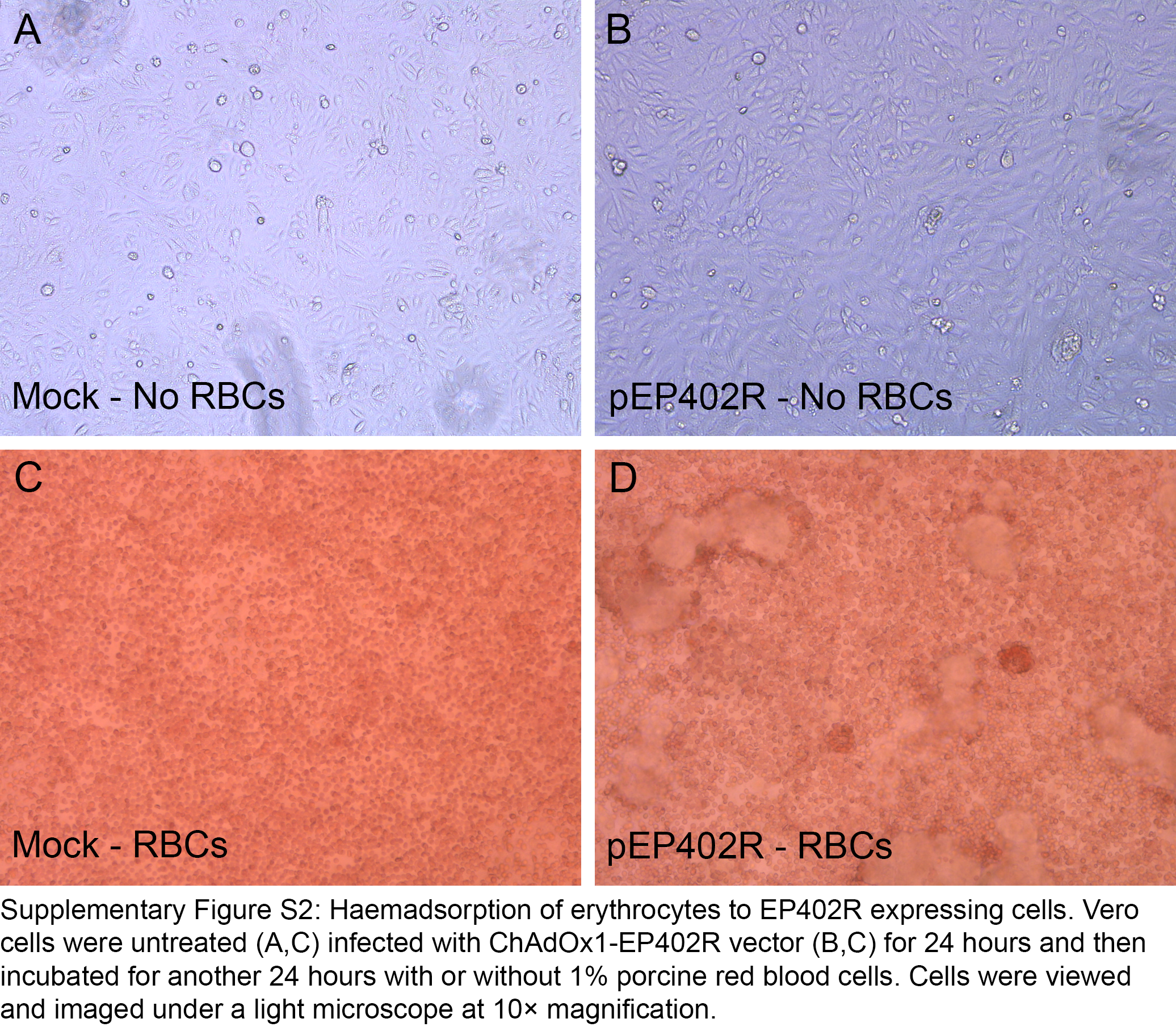

Supplement: Fig. S2 — Light microscopy images. [file spectrum.02328-25-s0003.tif]
