# Supplementary material for: African swine fever virus genes vectored by simian adenoviruses do not protect against virulent genotype II virus challenge
Source: Microbiol Spectr. 2026 Feb 27;14(4):e02328-25. doi: 10.1128/spectrum.02328-25 (PMC13055240; doi:10.1128/spectrum.02328-25)
Supplement: Supplemental material — Supplemental methods; Fig. S3 to S11. [file spectrum.02328-25-s0004.pdf]

Supplemental Material

African swine fever virus genes vectored by simian adenoviruses do not protect against virulent genotype II virus challenge.

Priscilla Y L Tng, Laila Al-Adwani, Lynnette Goatley, Eleni-Anna Loundras, Claire Powers, Christopher L Netherton

Supplementary Methods

Antibodies and anti-serum

**Target**	**Clone/Name**	**Species**	**Dilution**	**Reference**
ASFV	AW21	Pig	1:1000 (IF)	{Rathakrishnan, 2022 #1715}
V5	SV5-Pk1	Mouse	1:500 (IF)	Ab27671

Protein expression analysis

Vero cells were infected with 125 IU/cell of ChAdOx1 encoding ASFV genes. Cells were fixed with 4% paraformaldehyde for 30 minutes or lysed in RIPA sample preparation buffer 18 hour post infection with ChAdOx1. Transfected cells were also treated with 10 µM MG-132 for 16 hours prior to fixation. Cells for immunofluorescence were permeabilised with 0.2% Triton X-100 in PBS, incubated with blocking buffer (cal/mag free PBS, 0.2% (w/v) gelatin, 10% (v/v) normal goat serum). Primary and secondary antibodies were also diluted in blocking buffer, cells were washed with PBS between all stages. The coverslips were mounted onto glass slides with vectashield + DAPI mounting media.

Competitive ELISA

Antibodies to p30 (*CP204L* gene) were detected using the ID Screen® African Swine Fever Competition ELISA (Innovative Diagnostics, Grabels, France). Assays were run and positive, negative and inconclusive samples calculated according to the respective manufacturer's instructions.

Viral vectors

ChAdOx1 is a replication-deficient (E1 and E3 deleted) simian adenovirus, derived from Y25, that has been used extensively as a viral vector vaccine in both human and animal models. ChAdOx1 vectors were produced and cultured using T-REx™-293 cells (ThermoFisher Scientific), a derivative of HEK293 cells that both expresses the E1 proteins required for virus replication and constitutively express the Tet repressor protein to prevent antigen expression from a tetracycline regulated promoter during production. ChAdOx1-GFP has been described previously.

Codon-optimised genes were designed using amino acid sequences obtained from the Georgia 2007/1 isolate of ASFV (FR682468.2). The fusion protein was comprised of Sus scrofa ubiquitin C, I73R, F334L, CP204L, M448R, B646L with the sequence of a V5 epitope tag at the 3' end. Open reading frames were cloned into Gateway® shuttle vectors and then transferred to ChAdOx1 bacterial artificial chromosomes using standard methods. Linearised BAC DNA was transfected into a single 6 well of T-REx™-293 cells using lipofectamine 2000 in OptiMEM using standard methodology, with media changed after 4 hours to a standard growth media containing serum. After a few days, the transfected cells were passed into a 75cm2 flask and the resulting culture harvested 2 days later. Virus material was amplified by passage and virus material recovered from lysed cells. This material was used as a pre-master stock for standard preparations of adenovirus. ChAdOx1 titres were determined on T-REx™ cells using anti-hexon immunostaining assay based on the QuickTiter™ Adenovirus Titer Immunoassay kit (Cell Biolabs Inc.).

Clonal stocks of vectors encoding B602L, E183L, E199L, EP153R, EP402R, F317L, MGF505-5R, and O61R were prepared by infecting T-REx™-293 cells cultured in 96-well plates at a multiplicity of infection of 0.3. Plates were cultured for several days and wells showing cytopathic effect harvested and were sequentially cultured for 48 hours on 6 well plate swells followed by 75cm2 flasks. The presence of the transgene was confirmed by PCR and then process was repeated two more times to obtain stable clonal material of each vector.

Clonal stocks and ChAdOx1 expressing the ubiquitinylated fusion protein were used to infect bulk cultures of T-REx™-293 cells. These were harvested after 48h, lysed and purified by CsCl gradient as described previously (82). The resulting material was then buffer exchanged into a suitable storage solution, filter sterilised and aliquoted. Quality control checks were performed on the material, which included: sterility, viral particle (VP) analysis by spec, infectious unit quantification by immunostaining, and transgene assessment by PCR.

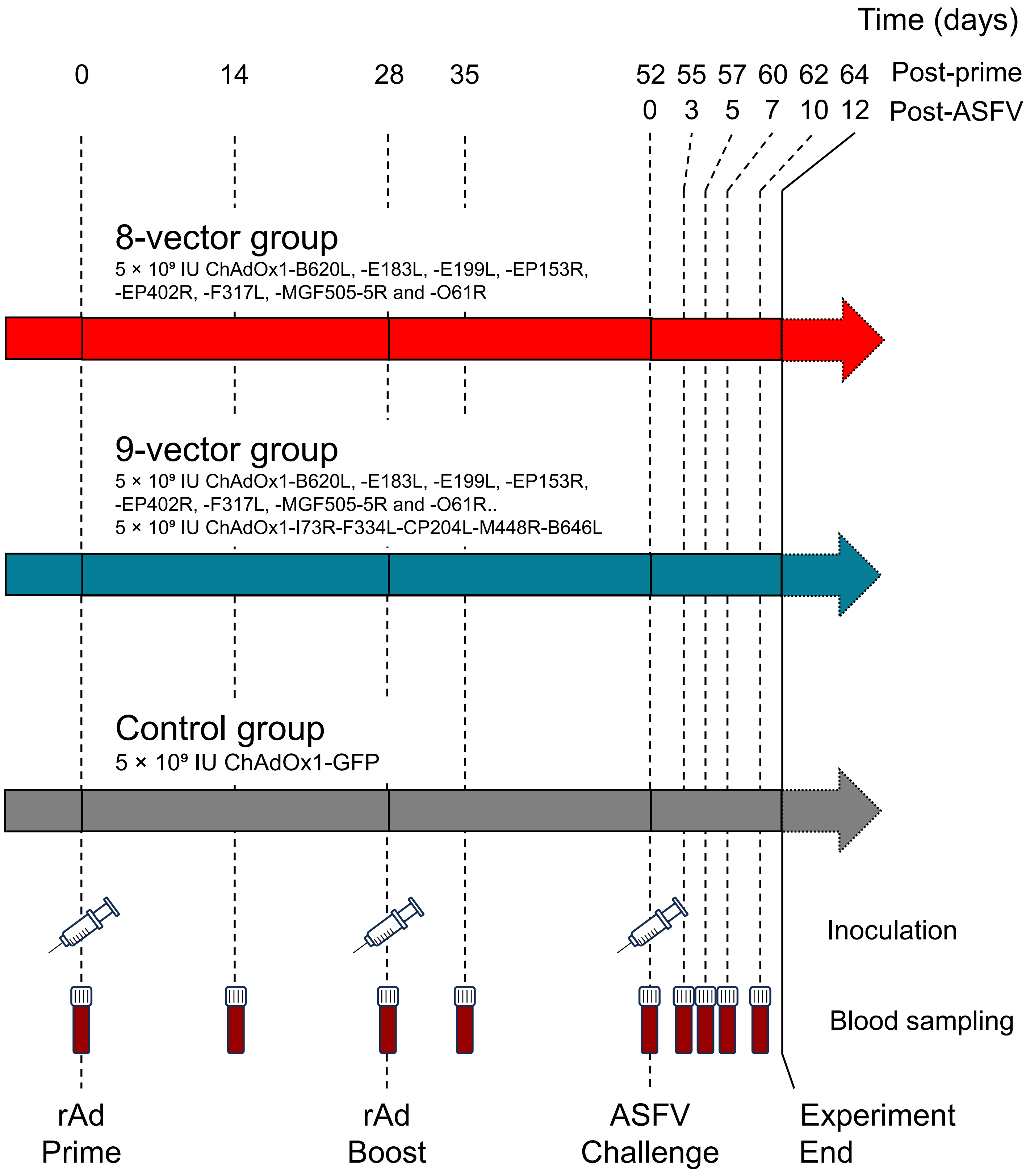

Supplementary Figure S3: Animal experiment timeline. Animals were randomly assigned to groups and allowed to acclimatize for seven days before inoculation via the intramuscular route with replication deficient ChAdOx1 adenoviruses expressing the indicated genes (prime). Pigs were given a second dose of the same adenoviruses 28 days after the prime (boost) and were inoculated with ASFV 52 days after the prime (challenge). Blood samples were taken, 0, 14, 28, 25, 52, 55, 57, 60 and 62 days after the first adenovirus immunisations from the surviving animals. Blood samples collected 52, 55, 57, 60, 62 and 64 days post prime were equivalent to 0, 3, 5, 7, 10 and 12 days post challenge. The experiment ended 64 days post prime/12 days post challenge.

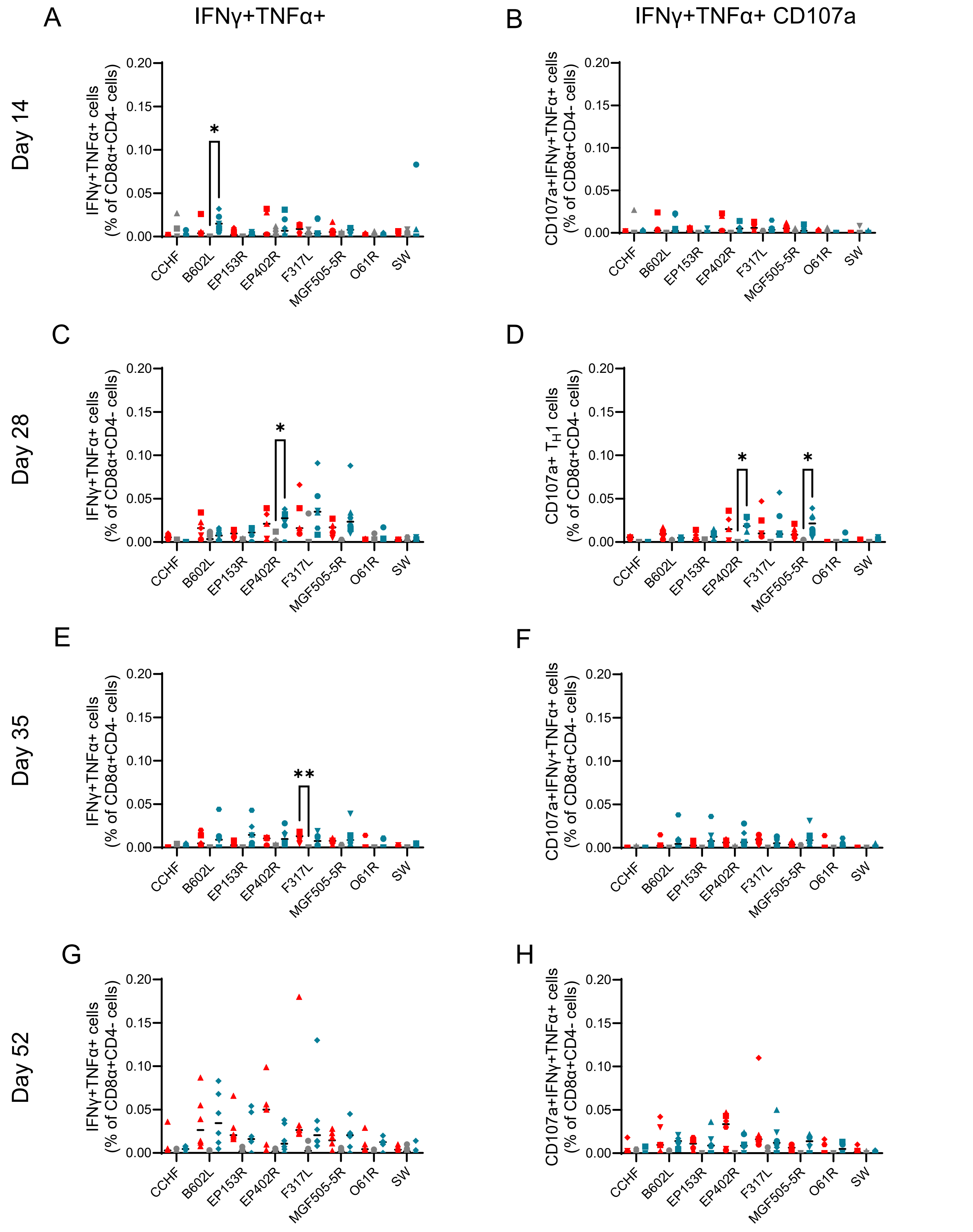

Supplementary Figure S4: Antigen specific CD8 T-cell responses. Animals were immunised with 8 vectors (red), GFP (grey) or 9 vectors (blue) and boosted 28 days later. PBMCs purified on the indicated days were stimulated with peptides pools corresponding to the indicated gene products or the ubiquitinylated polyprotein (SW).. The CD3+CD8α+CD4- population was identified by flow cytometry and the proportion of them expressing IFNγ and TNFα (A-D) or IFNγ, TNFα and CD107a (E-H) two weeks post prime (A, D), pre-boost (B, E), seven days post boost (C, F) and pre-challenge (D, H) determined. Bars indicated the means of each group and statistically significant differences between the groups shown.

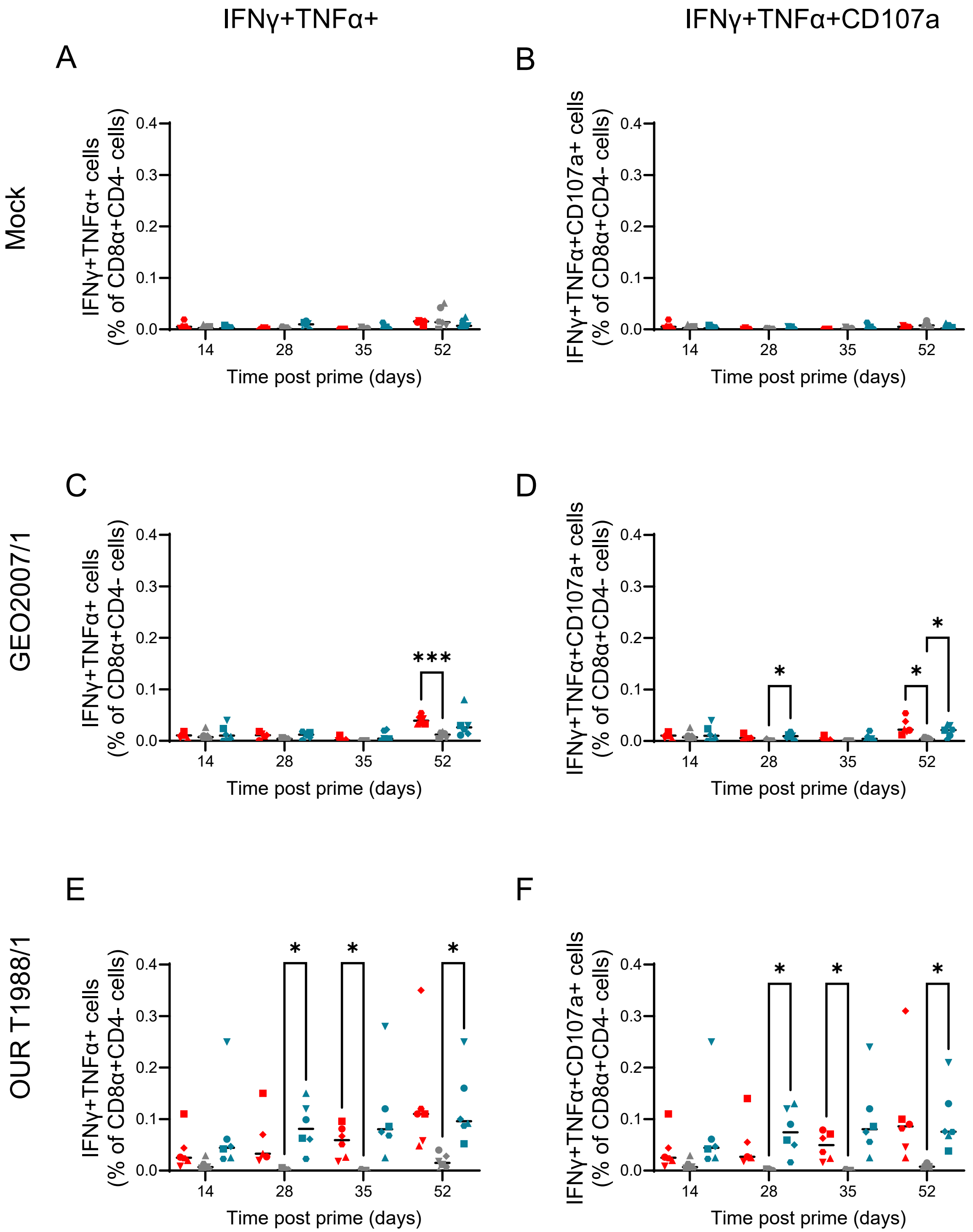

Supplementary Figure S5: Virus specific CD8 T-cell responses. Animals were immunised with 8 vectors (red), GFP (grey) or 9 vectors (blue). Cells were purified from blood collected on the indicated days and stimulated with mock (A-B), OUR T1988/1 (C-D) or Georgia 2007/1 (E-F) inocula overnight and then treated with brefeldin A and anti-CD107a for four hours. Populations of CD3+CD8α+CD4- cells were then identified by flow cytometry and the proportion of them expressing either IFNγ and TNFα (A, C, E), or IFNγ, TNFα and CD107a (B, D, F) determined. Bars indicated the means of each group and statistically significant differences between the groups shown.

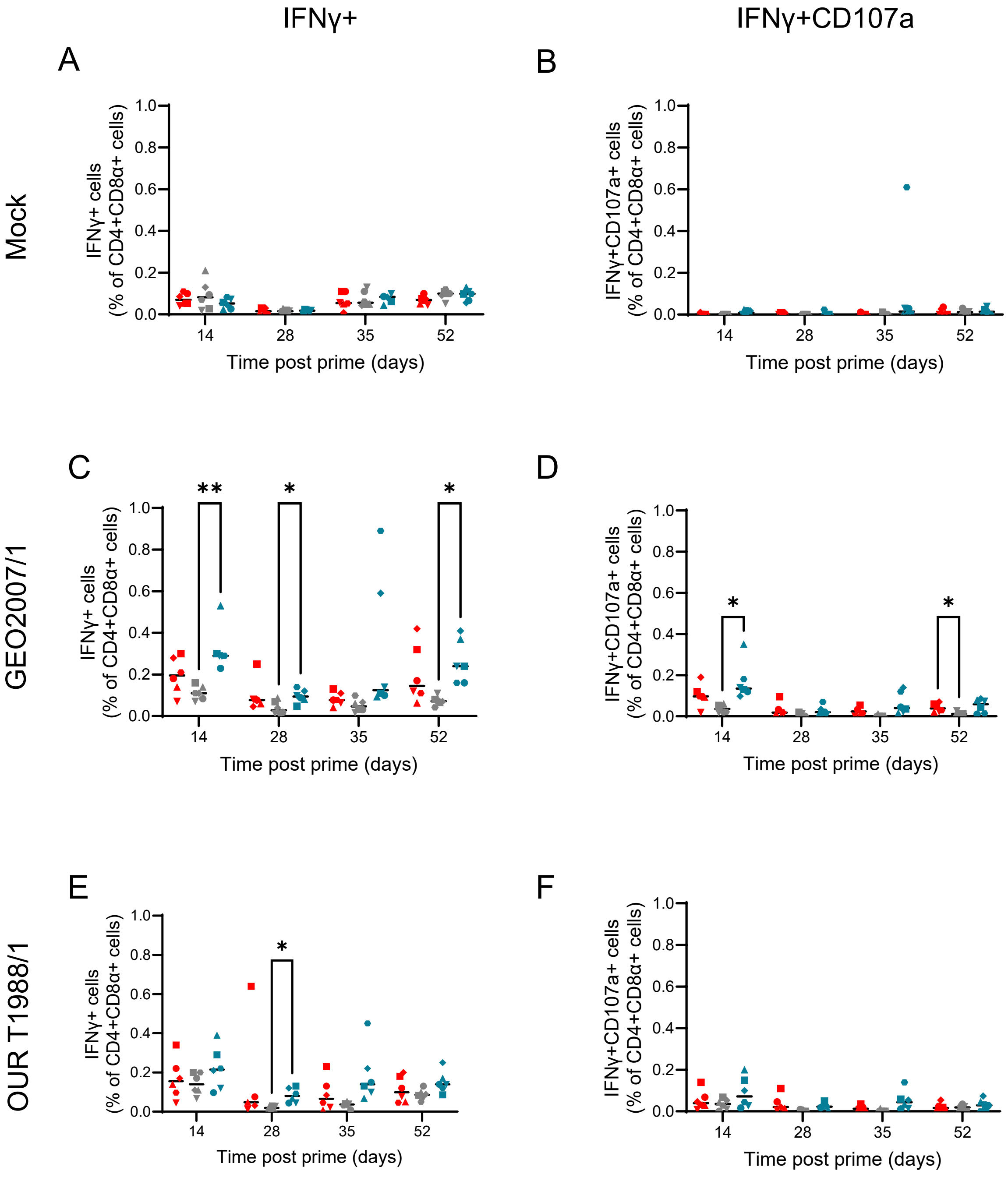

Supplementary Figure S6: Virus specific CD4 T-cell responses. Animals were immunised with 8 vectors (red), GFP (grey) or 9 vectors (blue). Cells were purified from blood collected on the indicated days and stimulated with mock (A-B), OUR T1988/1 (C-D) or Georgia 2007/1 (E-F) inocula overnight and then treated with brefeldin A and anti-CD107a for four hours. Populations of CD3+CD4+CD8α+ cells were then identified by flow cytometry and the proportion of them expressing either IFNγ (A, C, E), IFNγ and CD107a (B, D, F) determined. Bars indicated the means of each group and statistically significant differences between the groups shown.

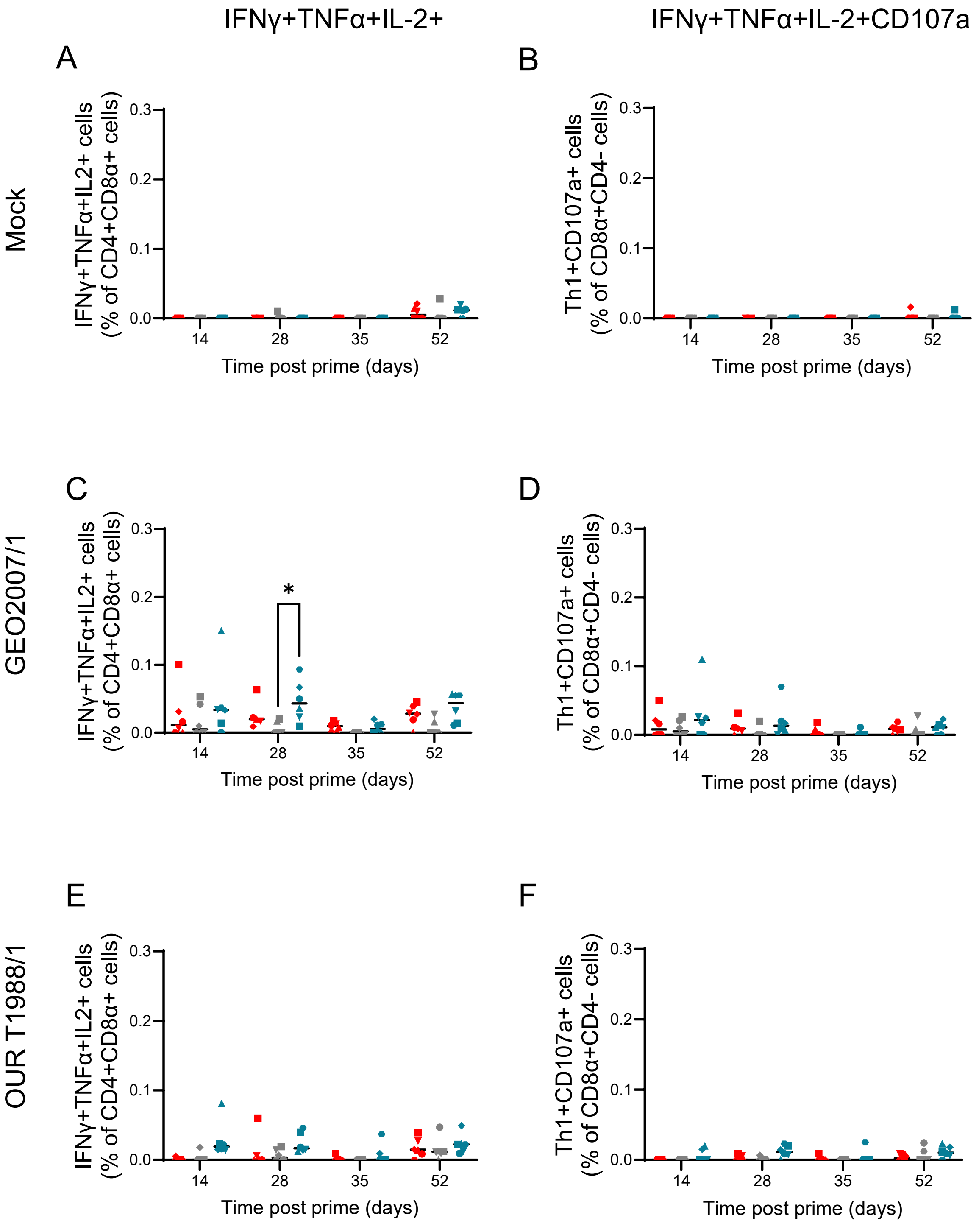

Supplementary Figure S7: Virus specific CD4 T-cell responses. Animals were immunised with 8 vectors (red), GFP (grey) or 9 vectors (blue). Cells were purified from blood collected on the indicated days and stimulated with mock (A-B), OUR T1988/1 (C-D) or Georgia 2007/1 (E-F) inocula overnight and then treated with brefeldin A and anti-CD107a for four hours. Populations of CD3+CD4+CD8α+ cells were then identified by flow cytometry and the proportion of them expressing either IFNγ, TNFα and IL-2 (A, C, E), or IFNγ, TNFα, IL-2 and CD107a (B, D, F) determined. Bars indicated the means of each group and statistically significant differences between the groups shown.

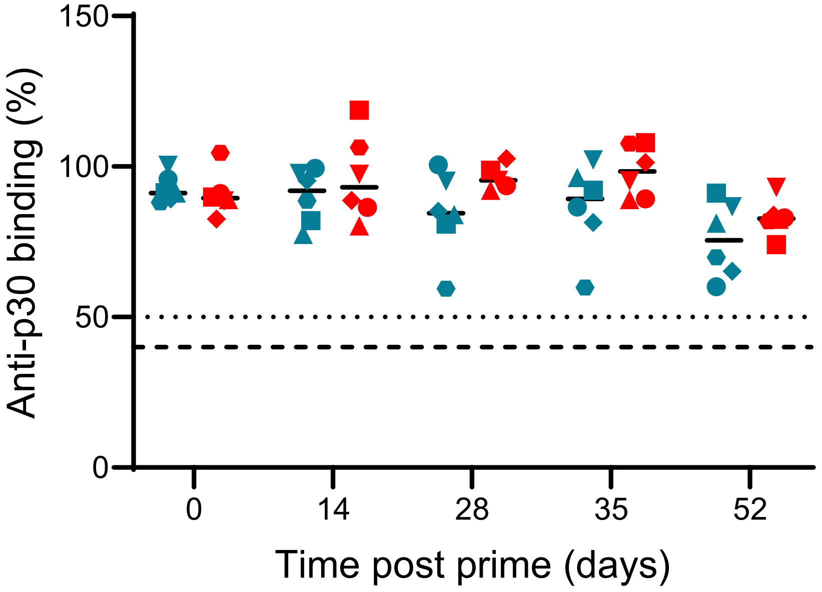

Supplementary Figure S8: P30 specific antibody responses. Animals were immunised with 8 vectors (red) or 9 vectors (blue), boosted 28 days later and then challenged on Day 52. Serum was collected on the indicated days and antibody responses against p30 determined by ID Screen® competition ELISA. The positive cutoff (≤ 40%) is indicated by a dashed line and the inconclusive cut off by a dotted line (> 40% < 50%). Bars indicated the means of each group.

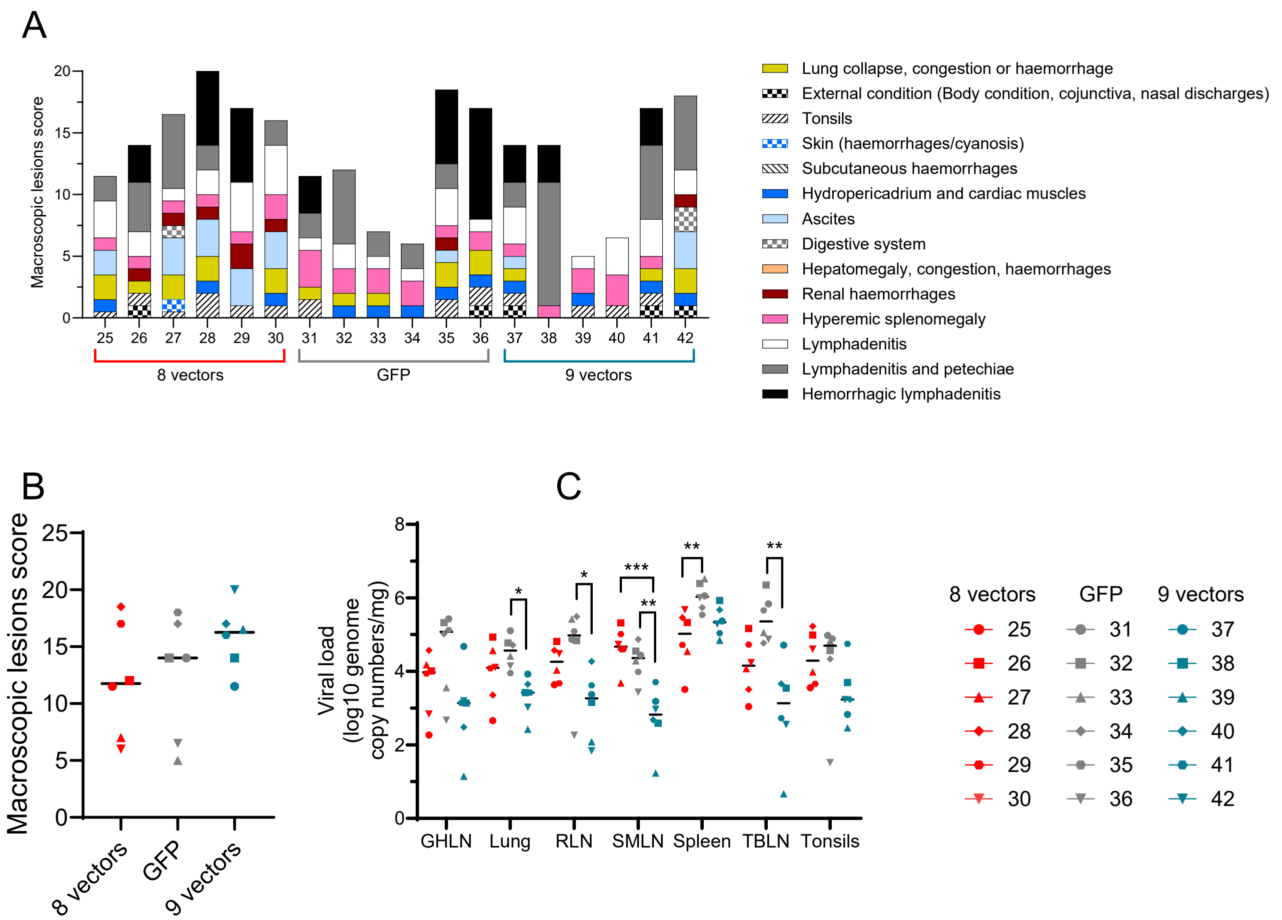

Supplemental Figure S9: Challenge data. Pigs immunised with 8 vectors (red), GFP (grey), or 9 vectors (blue) were challenged with ASFV Georgia 2007/1. Survival curves for each group (A) and mean clinical scores (B) were recorded. Animals were assessed for macroscopic lesions during necropsies (C,D). Viral load in tissues collected post mortem was determined by qPCR (E).

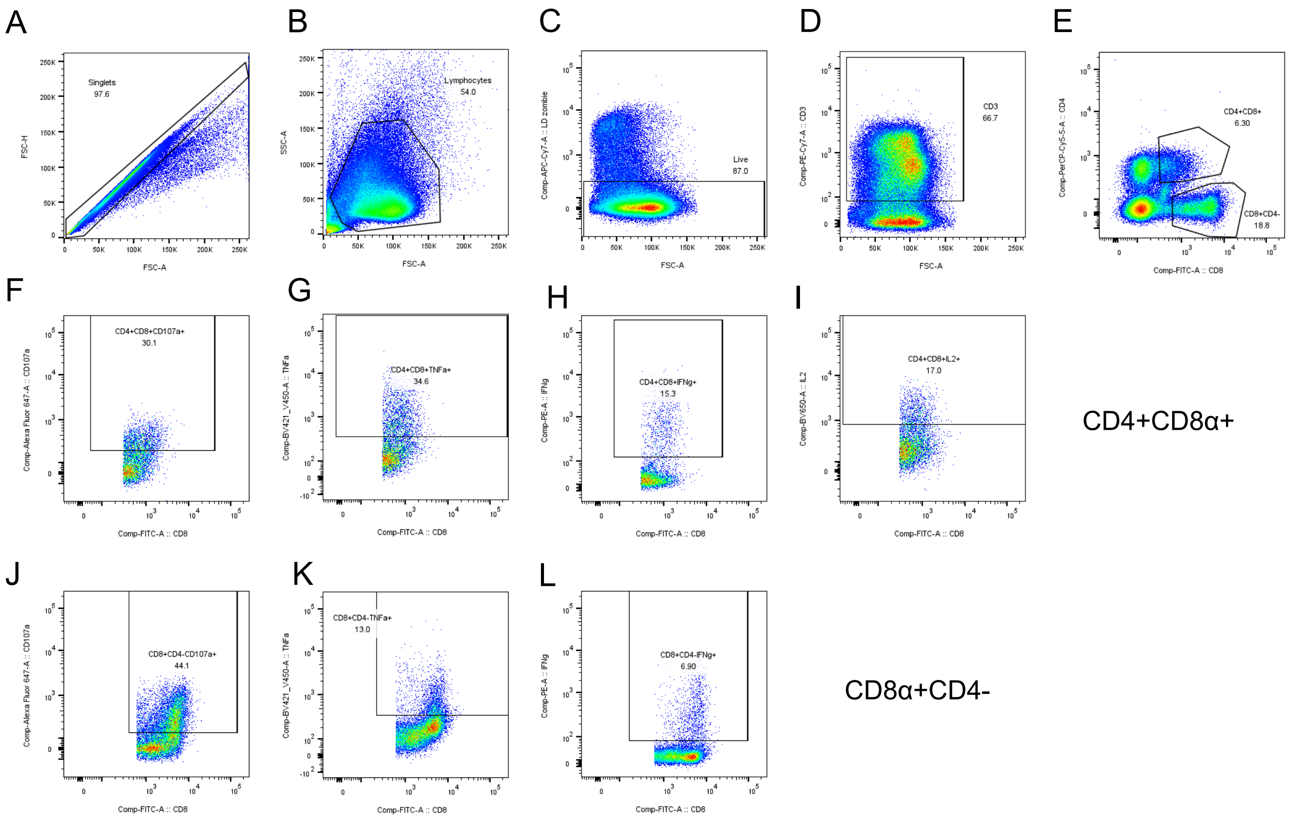

Supplementary Figure S10: Gating strategy for identifying virus and antigen specific responses by flow cytometry. PBMCs were stimulated with virus or antigens overnight and then treated with brefeldin A and anti-CD107a during the final four hours. Cells were stimulated with PMA/ionomycin for four hours after resting overnight. Cells were then incubated with Zombie NIR, labelled with anti-CD3, anti-CD4 and anti-CD8α before fixation. Cytokines were detected using anti-IFNγ, anti-TNFα and anti-IL-2. Gates were used to identify single cells (A), lymphocytes (B), followed by live cells (C) which were then divided into CD3+ and CD3- populations (D). CD4+CD8α+ and CD8α+CD4- (E) were gated from the CD3+ population and then the numbers of CD107a+ (F,J), TNFα+ (G,K) and IFNγ+ (H,L) cells identified from both populations, as well as the number of IL-2+ cells (I) from the CD4+CD8α+ population. Pre-challenge (Day 52) cells collected from Pig 41 and simulated with PMA/ionomycin are shown.

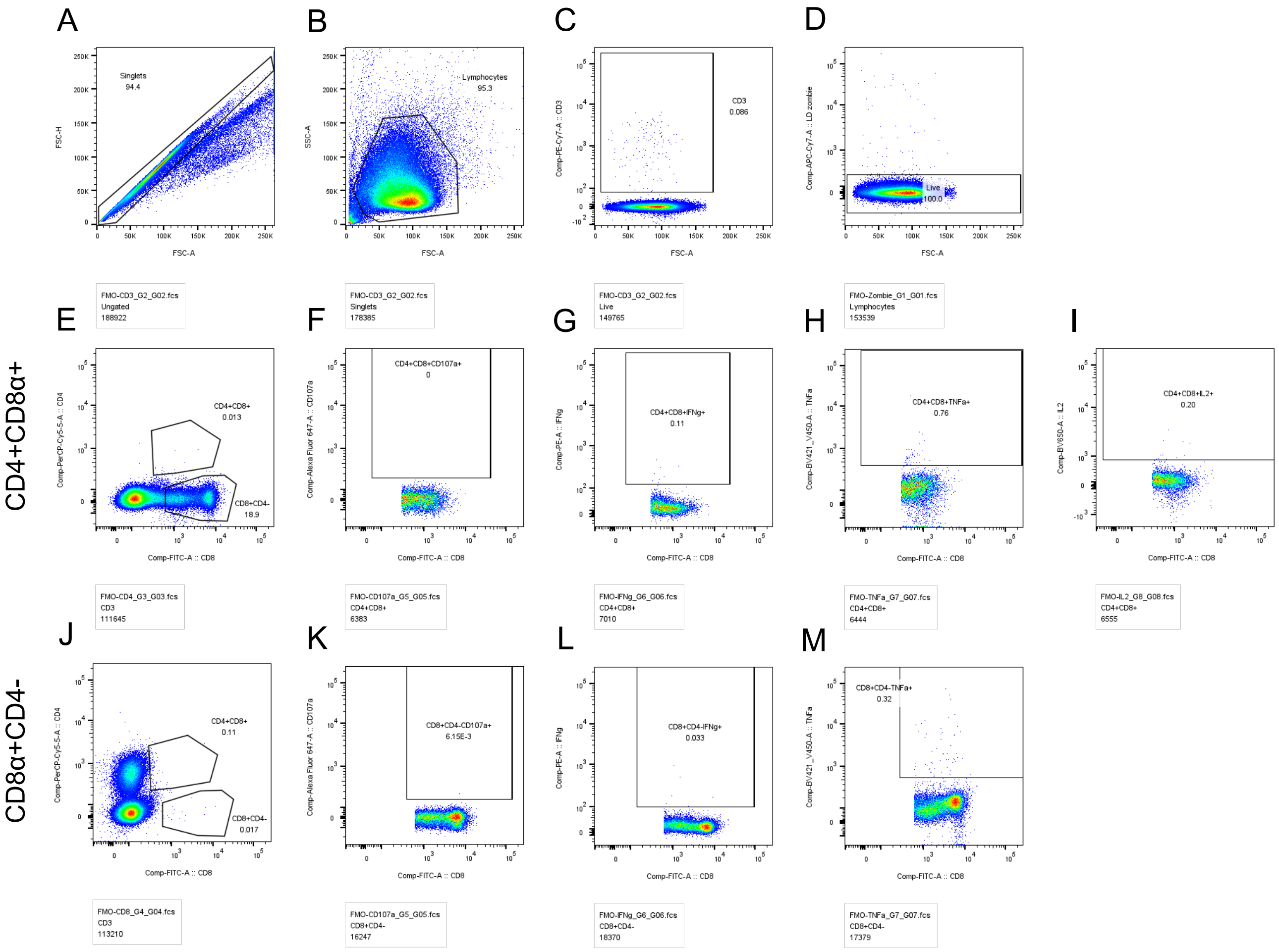

Supplementary Figure S11: Fluorescence minus ones (FMOs). Cells were treated, stained and gated to define single lymphocytes (A, B) as described in the legend for Supplementary Figure S9, except that the following dyes or antibodies were omitted. CD3-PECy7 (C), Zombie Near Infrared (D), CD4-PerCpCy5.5 (E), CD107a-AF647 (F, K), IFNγ-PE (G, L), TNFα-BV421 (H, M), IL-2-BV650 (I) or CD8α-FITC (J). Pre-challenge (Day 52) cells collected from Pig 41, simulated with PMA/ionomycin and gated on live CD3+CD4+CD8α+ (E to I) and live CD3+CD8α+CD4- (J to M) cells are shown.
